# The complete European guidelines on phenylketonuria: diagnosis and treatment

**DOI:** 10.1186/s13023-017-0685-2

**Published:** 2017-10-12

**Authors:** A. M. J. van Wegberg, A. MacDonald, K. Ahring, A. Bélanger-Quintana, N. Blau, A. M. Bosch, A. Burlina, J. Campistol, F. Feillet, M. Giżewska, S. C. Huijbregts, S. Kearney, V. Leuzzi, F. Maillot, A. C. Muntau, M. van Rijn, F. Trefz, J. H. Walter, F. J. van Spronsen

**Affiliations:** 1 Division of Metabolic Diseases, Beatrix Children’s Hospital, University Medical Center Groningen, PO BOX 30.001, 9700 RB Groningen, The Netherlands; 2 Dietetic Department, Birmingham Children’s Hospital, Birmingham, UK; 3 Department of PKU, Kennedy Centre, Glostrup, Denmark; 4 Metabolic Diseases Unit, Department of Paediatrics, Hospital Ramon y Cajal Madrid, Madrid, Spain; 5 University Children’s Hospital, Dietmar-Hoppe Metabolic Centre, Heidelberg, Germany; 6 University Children’s Hospital Zürich, Zürich, Switzerland; 7 Department of Paediatrics, Division of Metabolic Disorders, Academic Medical Centre, University Hospital of Amsterdam, Amsterdam, The Netherlands; 8 Division of Inherited Metabolic Diseases, Department of Paediatrics, University Hospital of Padova, Padova, Italy; 9 Neuropaediatrics Department, Hospital Sant Joan de Déu, Universitat de Barcelona, Barcelona, Spain; 10 Department of Paediatrics, Hôpital d’Enfants Brabois, CHU Nancy, Vandoeuvre les Nancy, France; 11 Department of Paediatrics, Endocrinology, Diabetology, Metabolic Diseases and Cardiology of the Developmental Age, Pomeranian Medical University, Szczecin, Poland; 12 Department of Clinical Child and Adolescent Studies-Neurodevelopmental Disorders, Faculty of Social Sciences, Leiden University, Leiden, The Netherlands; 13 Clinical Psychology Department, Birmingham Children’s Hospital, Birmingham, UK; 14 Department of Paediatrics, Child Neurology and Psychiatry, Sapienza University of Rome, Via dei Sabelli 108, 00185 Rome, Italy; 15 CHRU de Tours, Université François Rabelais, INSERM U1069, Tours, France; 16 University Children’s Hospital, University Medical Centre Hamburg-Eppendorf, 20246 Hamburg, Germany; 17 Department of Paediatrics, University of Heidelberg, Heidelberg, Germany; 18 Medicine, Manchester Academic Health Sciences Centre, Central Manchester University Hospitals NHS Foundation Trust, Manchester, UK

**Keywords:** European, Guidelines, Phenylalanine hydroxylase deficiency, PAH deficiency, Phenylketonuria, PKU, Hyperphenylalaninemia, Phenylalanine, Treatment, Management, Recommendations, Tetrahydrobiopterin, Sapropterin

## Abstract

Phenylketonuria (PKU) is an autosomal recessive inborn error of phenylalanine metabolism caused by deficiency in the enzyme phenylalanine hydroxylase that converts phenylalanine into tyrosine. If left untreated, PKU results in increased phenylalanine concentrations in blood and brain, which cause severe intellectual disability, epilepsy and behavioural problems. PKU management differs widely across Europe and therefore these guidelines have been developed aiming to optimize and standardize PKU care. Professionals from 10 different European countries developed the guidelines according to the AGREE (Appraisal of Guidelines for Research and Evaluation) method. Literature search, critical appraisal and evidence grading were conducted according to the SIGN (Scottish Intercollegiate Guidelines Network) method. The Delphi-method was used when there was no or little evidence available. External consultants reviewed the guidelines. Using these methods 70 statements were formulated based on the highest quality evidence available. The level of evidence of most recommendations is C or D. Although study designs and patient numbers are sub-optimal, many statements are convincing, important and relevant. In addition, knowledge gaps are identified which require further research in order to direct better care for the future.

## Background

**Fig. 1:**
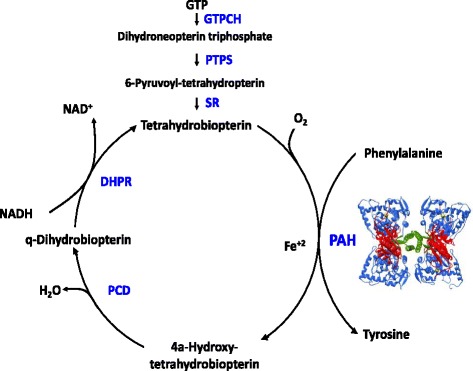
Phenylalanine hydroxylating system. BH4: tetrahydrobiopterin; DHPR: dihydropteridine reductase; GTP: guanosine triphosphate; GTPCH: GTP cyclohydrolase I; Phe: Phenylalanine; PAH: phenylalanine hydroxylase; PCD: phenylalanine carbinolamie-4a-dehydratase; PTPS: 6-pyruvoyl-tetrahydropterin synthase; SR: sepiapterin reductase

**Fig. 2:**
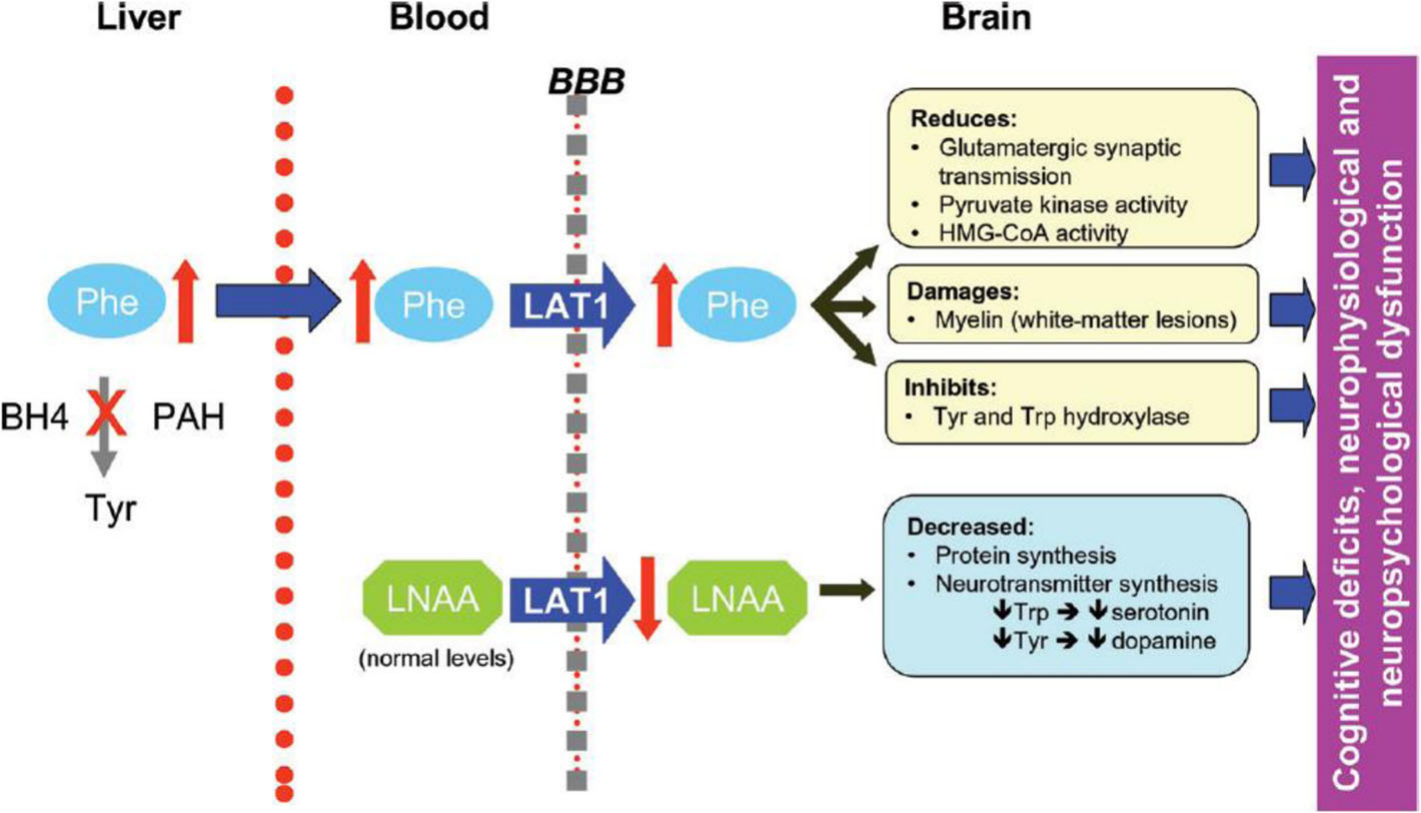
Pathophysiology of PKU: Summary of potential mechanisms of neurocognitive impairment by high phenylalanine concentrations. Phe: phenylalanine; BBB, blood–brain barrier; LNAA: Large Neutral Amino Acids; LAT1, L-type amino acid carrier; BH4, tetrahydrobiopterin; HMG-CoA, 3-hydroxy-3-methylglutaryl-coenzyme A; Tyr, tyrosine; Trp, tryptophan

Phenylketonuria (PKU; McKusick #261600) is a rare autosomal recessive inborn error of phenylalanine (Phe) metabolism caused by variants in the gene encoding phenylalanine hydroxylase (PAH). PAH normally converts Phe into tyrosine (Tyr) requiring the cofactor tetrahydrobiopterin (BH4), molecular oxygen and iron (Fig. 1) [1]. PAH deficiency leads to accumulation of Phe in the blood and brain. Untreated, PKU is characterized by irreversible intellectual disability, microcephaly, motor deficits, eczematous rash, autism, seizures, developmental problems, aberrant behaviour and psychiatric symptoms. The precise pathogenesis of brain dysfunction is still unclear (Fig. 2) [2]. As high blood Phe concentrations are strongly related to neurocognitive outcome, existing treatments aim at decreasing blood Phe concentrations. PKU was identified in 1934 by Følling when he detected phenylketone bodies in the urine of affected individuals and in 1953, Bickel first reported the effectiveness of a low-Phe diet in a child with PKU. In the 1960’s, Guthrie developed a simple test to detect hyperphenylalaninemia (HPA) in large populations. This led to PKU becoming the first disorder to benefit from newborn screening; its early detection and treatment prevented mental retardation. However, the NBS screen is for HPA and this is defined as any blood Phe >120 μmol/L. Therefore, in every positive NBS for Phe, primary phenylalanine hydroxylase deficiency should be distinghuished from other causes of HPA including pterin defects, high protein intake, liver disease or HPA not requiring treatment. This guideline is for PKU and does not discuss pterin defects which necessitate different treatment and follow-up [3].

The prevalence of PKU varies worldwide. In Europe, the mean prevalence is approximately 1:10,000 newborns with a higher rate in some countries such as Ireland and Turkey, and a very low rate in Finland [4].

Due to NBS and treatment commencement shortly after birth, patients fall within the broad normal range of general ability, attain more or less expected educational standards and lead independent lives as adults. As a consequence, PKU is considered a medical success story but neuropsychological deficits, behavioural and social issues occur in some patients, and (as a group) their mean neurocognitive always level is somewhat below their siblings or control groups from the general population [1, 5].

The cornerstone of PKU treatment is a low Phe diet in combination with Phe-free L-amino acid supplements. Some PKU centres use casein glycomacropeptide (GMP) or large neutral amino acids (LNAA) as alternative dietary supplements. Certain patients are responsive to and are treated with BH4, acting as a pharmaceutical chaperone (prescribed as sapropterin dihydrochloride) [1]. Possible future treatments include enzyme substitution and gene therapy.

PKU management differs widely across Europe, even though the evidence on which management is based is the same [6–8]. Therefore, the development of European PKU guidelines was considered necessary [8–10] and initiated after the publication of the consensus paper by the European Society of Phenylketonuria and Allied Disorders (ESPKU) [11]. Guidelines can result in measurable improvements in patient care [12, 13], provision of consistent, high-quality treatment without inequality, and rare disease awareness [14]. The key statements from this guideline were published recently [15]. The difficulty in rare disease guideline development is that high quality studies that include large patient numbers are scarse. Evidence is lacking in several areas including treatment initiation and adult management goals. Therefore, guidelines may change when new data is available. The goal of these European guidelines is to offer a standard for diagnostics, treatment and care in PKU that would lead to optimal clinical and neuropsychological outcome without overtreatment and unnecessary costs. These guidelines are intended to be used by metabolic physicians, dieticians, obstetricians, midwives, psychologists, social workers, biochemists and other professionals involved in the treatment of patients with PKU due to PAH deficiency.

## Methods

The scientific advisory committee of the ESPKU was asked to invite a group of European PKU experts based on their expertise and experience rather than their nationality. Nineteen were invited; 1 declined and 1 resigned for personal reasons. The 17 remaining professionals were divided into 5 working groups and supported by a project lead (F.J. van Spronsen) and project assistant (A.M.J. van Wegberg). Working group members included 8 paediatric metabolic physicians, an adult metabolic physician, 2 paediatric neurologists, 1 biochemist, 3 metabolic dieticians and 2 (neuro) psychologists. Some assisted more than 1 working group and an obstetrician was consulted by the maternal PKU group. These guidelines were developed between October 2012 and December 2015.

The Appraisal of Guidelines for Research and Evaluation (AGREE) method was used to formulate the guidelines. The literature search, critical appraisal and evidence grading were performed according to the Scottish Intercollegiate Guidelines Network (SIGN) method version 2011 (http://www.sign.ac.uk/) (Table 1). There was one update (version 2014) as SIGN decided not to continue with the ABCD grading. At the start of these guidelines, development version 2011 was the appropriate methodology. Forthcoming updates will use the new GRADE process.

**Table 1:** SIGN grading system 1999–2012

Levels of evidence	
1++	High quality meta-analyses, systematic reviews of RCTs, or RCTs with a very low risk of bias
1+	Well-conducted meta-analyses, systematic reviews, or RCTs with a low risk of bias
1-	Meta-analyses, systematic reviews, or RCTs with a high risk of bias
2++	High quality systematic reviews of case control or cohort or studies High quality case control or cohort studies with a very low risk of confounding or bias and a high probability that the relationship is causal
2+	Well-conducted case control or cohort studies with a low risk of confounding or bias and a moderate probability that the relationship is causal
2-	Case control or cohort studies with a high risk of confounding or bias and a significant risk that the relationship is not causal
3	Non-analytic studies, e.g. case reports, case series
4	Expert opinion
Grades of recommendations	
	At least one meta-analysis, systematic review, or RCT rated as 1++, and directly applicable to the target population; *or* A body of evidence consisting principally of studies rated as 1+, directly applicable to the target population, and demonstrating overall consistency of results
	A body of evidence including studies rated as 2++, directly applicable to the target population, and demonstrating overall consistency of results; or Extrapolated evidence from studies rated as 1++ or 1+
	A body of evidence including studies rated as 2+, directly applicable to the target population and demonstrating overall consistency of results; *or* Extrapolated evidence from studies rated as 2++
	Evidence level 3 or 4; *or* Extrapolated evidence from studies rated as 2+
Good practice points	
	Recommended best practice based on the clinical experience of the guideline development group

The 5 working groups defined key questions on the following 6 subjects: 1) Nutritional treatment and biochemical/nutritional follow up; 2) Neurocognitive outcome including imaging, psychosocial outcome and adherence; 3) Adult and maternal PKU; 4) Late diagnosed and untreated PKU; 5) Diagnosis of PKU including treatment initiation; and 6) Pharmacological treatment of PKU. They searched for relevant literature in PubMed (MEDLINE), EMBASE, NHS Economic Evaluations Database and The Cochrane Library being helped by the project assistant. For some subjects, additional search systems were used and reference lists were checked. All reviewed literature was published before Dec 31, 2015 and did not exclude any publications before a specified year or type of study design. Papers were excluded if they were not relevant to the key question or not written in English language. A total of 975 publications was reviewed. The methodological quality of the studies was assessed by 2 group members independently and/or by group discussion. Recommendations were either based on evidence (if level of evidence was A or B using the SIGN method) or by consensus using the Delphi method (if the level of evidence was C, D or the so-called good practice points that are not based on any evidence). To reach such consensus, those recommendations without high level of evidence were discussed with all participants of all working groups during 5 face-to-face plenary sessions using Delphi methodology. All working groups and plenary sessions were facilitated by the guidelines lead and/or the project assistant.

Because of the rarity of this disorder, there were limited high quality papers available for most subjects, even though PKU is one of the most researched inherited metabolic disorders (IMD). Most papers described cohort/chart studies, cross-sectional or descriptive studies, and therefore, most subjects and evidence did not exceed level C. Although the design of many studies was sub-optimal or they lacked statistical power, the statements written in this guideline are convincing, important and relevant.

Consistency, applicability and volume of evidence were considered with some evidence upgraded or downgraded accordingly. There was no grading system available for diagnostic accuracy evidence.

A concept of the guideline was sent to 16 external consultants specialized in PKU management. Fifteen of them responded, while 2 reviewers chose to remain anonymous; S. Beblo (Germany), G. Berry (US), M. Bik-Multanowski (Poland), M. Cleary (United Kingdom), T. Coşkun (Turkey), H. Gökmen-Özel (Turkey), J. Häberle (Switzerland), R. Lachmann (United Kingdom), H. Levy (United States), Y. Okano (Japan), I. Schwartz (Brazil), J. Zeman (Czech Republic), and patient organization ESPKU.

For subjects where the evidence was unconvincing, this may be translated into daily practice as either: 1) no treatment/impact of guidelines until proven to be effective, or 2) treatment/implementation until proven otherwise.

A grant was received from the ESPKU to fund a project assistant. The ESPKU or other people outside the guideline team had no opportunity to influence the development of the guideline statements or the full guideline document (except the 14 professionals and the ESPKU when invited to provide their external review).

## Key recommendations

The following recommendations were highlighted as the key clinical recommendations that should be prioritized for implementation [15]. The grade of recommendation relates to the scientific evidence and does not reflect the clinical importance.
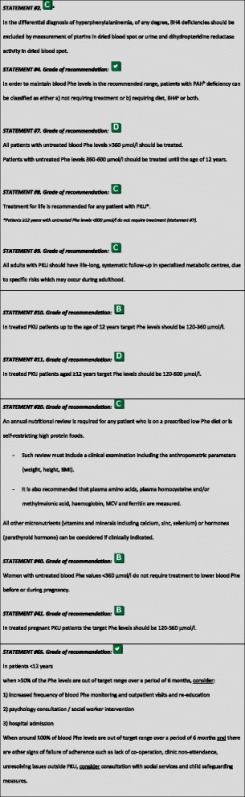


The marks range from V (no possibility to evaluate the level of evidence due to lack of any paper on this issue) to as high as B. ^a^In statement #2, a C level of evidence is chosen because of the high number of data notwithstanding that most included papers are of descriptive nature; ^b^PAH: phenylalanine hydroxylase; ^c^BH4: tetrahydrobiopterin; ^d^Phe: phenylalanine.

## Diagnosis

### Diagnosis

Published evidence confirms that universal NBS for PKU meets all accepted screening criteria and justifies the cost and infrastructure necessary for the collection and testing of neonatal blood spots [16–18]. NBS is considered a national obligation even in countries when populations are known not to have PKU. Due to high migration in countries, a diagnosis of PKU remains possible. NBS requires: 1) a robust infrastructure in which blood is taken from all newborns (ideally between 24 and 72 h after birth (Collaborative Laboratory Integrated Reports at http://clir.mayo.edu), to ensure timely start of treatment; and 2) a well-equipped laboratory that can handle bloodspots efficiently. Low-income countries may consider using the NBS laboratory facilities of other countries.

There are numerous committees and working groups that work on optimization of NBS procedures from the time of blood sampling, the method chosen for diagnosing high blood Phe levels and the referral procedure. At least partly, these procedures depend on national health care organizations. The most important issue is that children with a positive NBS result should be referred to a specialized metabolic centre with knowledge and experience in the diagnostic procedures and early treatment strategies to ensure the best outcome of PKU patients.

Individuals who have not had NBS and present with developmental delay or other PKU-related symptoms, should have plasma amino acids analysed.
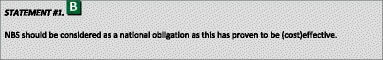


### Differential diagnosis of BH4 deficiencies

The differential diagnosis of HPA includes high natural protein intake, prematurity, defects in BH4 metabolism and liver disease. Patients with disorders of BH4 metabolism including GTP cyclohydrolyase I (GTPCH) deficiency, 6-pyruvoyl-tetrahydropterin synthase (PTPS) deficiency, dihydropteridine reductase (DHPR) deficiency and pterin-4a-carbinolamine dehydratase (PCD) deficiency can present with any degree of HPA [19, 20]. Some patients with GTPCH deficiency have normal Phe concentrations during the neonatal period [20, 21]. Dopa responsive dystonia caused by the dominant form of GTPCH deficiency and sepiapterin reductase (SR) deficiency [22] are not associated with HPA. With the exception of DHPR deficiency, which can be detected by determination of DHPR activity in dried blood spots (DBS), all other forms of BH4 deficiency (GTPCH, PTPS, and PCD deficiency) can be detected by specific pterin patterns in urine or DBS [19, 23, 24].

In cases where there may be delayed results of pterin and DPHR analysis, a 24-h BH4 loading test can be performed, in addition to analysis of pterins and DHPR that would allow earlier diagnosis of BH4-responsive PKU patients and/or BH4 deficiencies. Samples of blood and urine should be taken prior to starting treatment and before BH4 loading. Urine should be sampled and stored in dark conditions (by wrapping in aluminium foil) and stored immediately in a freezer. A useful alternative could be the use of next-generation sequencing panels [25, 26], but this methodology is only advisable when costs are lower and results are available within 7 days. Early diagnosis of GTPCH, PTPS and DHPR deficiencies may prevent irreversible brain damage by pharmacological treatment [20]. Those with PCD deficiency may be at risk of developing non-immune MODY-like diabetes or hypomagnesaemia and renal magnesium wasting [27, 28]. Evaluation for BH4 disorders for any neonate or infant with neurological problems of unknown origin is suggested even without increased Phe or negative NBS for increased Phe.
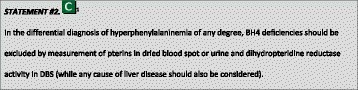


^1^Although most included papers are of descriptive nature the level of evidence is chosen to be C because of the high number of data.

### Genotyping

The gene encoding PAH is located on chromosome 12 (region q22–24.1) consisting of 13 exons and 12 introns, covering a total of 100 kb of genetic data. Over 950 *PAH* variants (*PAH*vdb database; http://www.biopku.org/home/pah.asp; last accessed 07–12-2015) are known to be associated with PAH deficiency. The majority of the variants (60%) are missense, usually resulting in protein misfolding and/or impairment of catalytic functions.

Patient genotyping is not essential for the diagnosis of PKU but the genotype can determine the degree of protein dysfunction, residual PAH activity and consequently the metabolic phenotype. The classification of PAH genotypes may allow for prediction of the biochemical and metabolic phenotypes in many genotypes and be useful for the management of HPA in newborns [29–32]. Also, at least to some degree, BH4-responsiveness may be predicted or excluded from the patient’s genotype [32–34]. Patients with gene variants that determine a high residual enzyme activity (which are those with the milder metabolic phenotypes) have a higher probability of responding to BH4 [35, 36]. Alleles that are known to be responsive to treatment with BH4 are listed in the BIOPKU database http://www.biopku.org/home/biopku.asp. Patients with a genotype known to be non-BH4-responsive should not undergo BH4 testing, while patients with a genotype with 2 BH4-responsive variations may directly proceed to a treatment trial rather than a BH4 loading test. In all other patients, a BH4 loading should be considered.

Prenatal diagnosis for PKU is feasible and genetic counselling depends on many issues including ethical, religious and legal issues in each country.
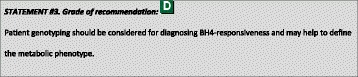


### PKU classification

There is no consensus regarding phenotype classification. Blaskovics developed a Phe loading test to differentiate subtypes based on the responses among 8 HPA disease types of which 5 were related to PAH deficiency [37]. However, at present, this is not regarded as ethical as it increases the Phe level. In 1980, untreated Phe levels, e.g., those measured at clinical diagnosis, were used by Güttler for PKU phenotyping [38]. These criteria no longer aid in diagnosing patients for various reasons, including the large range of cut-off points [39] and even more importantly, the time of neonatal screening, as patients will commonly start treatment before reaching their maximal Phe concentrations [40]. Additionally, Phe tolerance is used to differentiate among 3 or 4 phenotypes [38, 41]. Exact Phe tolerance is difficult to determine because of non standardized conditions and discrepancies between prescribed and actual intake of Phe.Therefore, the following simplified classification scheme is suggested, derived from Blau [3].
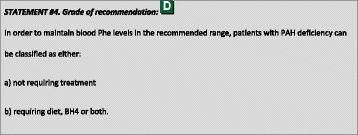


## Initiation of treatment and treatment for life

### Initiation of treatment

In 1990, Smith et al. showed that every 4 weeks’ delay in starting treatment caused a decline of IQ score by approximately 4 points [42], underscoring the knowledge that neurological damage starts early after birth. Although there are no formal studies to indicate that treatment commencement even earlier is necessary, data show that treatment in the early years of life has more impact than later years. As a consequence it is generally recommended that treatment should start as early as possible to prevent neurological damage [1]. We consider that treatment should be initiated before the age of 10 days, which for many countries will require change in timing of national NBS, logistical and diagnostic procedures.

There is unanimity in the literature and among professionals that patients with untreated blood Phe concentrations >600 μmol/l should be treated.

Except for the publication by Gassio et al. [43], no study has investigated if patients with untreated blood Phe levels <360 μmol/l should be treated. There is consensus that patients with untreated blood Phe levels <360 μmol/l should remain untreated, as this is not considered to be indicative of disease. Gassio et al. [43] found that individuals with HPA but with Phe levels <360 μmol/l without treatment, had scores on neuropsychological testing similar to control individuals except for 1 out of 2 executive function (EF) tests. However, this could also be explained by HPA patients having a lower average age than the control patients.

Because of the possibility of blood Phe concentrations increasing with age, patients with Phe levels <360 μmol/l should be monitored (at a lower frequency) during the first year of life as a minimum [44, 45].

The evidence regarding initiation of treatment with blood Phe concentrations between 360 and 600 μmol/l is inconsistent. Campistol et al. [39] and van Spronsen [46] discussed this dilemma. Costello et al. [47] found a trend towards lower intelligence quotient (IQ) in those with higher Phe levels when comparing 3 groups (<400, 400–500 and >500 μmol/l) and recommended treatment to maintain Phe <400 μmol/l throughout childhood in all forms of PKU. It was predicted that for every 100 μmol/L increase in mean Phe that IQ would decrease by approximately 6 IQ points. However, the groups were very small (*n* = 6, *n* = 11, and *n* = 7 respectively) and the paper had some methodological weakness as the study included patients with untreated Phe concentrations >600 μmol/l. Diamond et al. [48] observed that 10 children with untreated Phe levels between 360 and 600 μmol/l did not perform as well as healthy control children, although this was not statistically significant. However, their mean Phe during the first month of life was 900 μmol/l which is also considered a methodological flaw. In 2001, Weglage et al. studied 31 patients with untreated blood Phe levels between 360 and 600 μmol/l [49]. This data showed normal neuropsychological outcome data, but only a small number of patients (*n* = 7) had untreated Phe levels in the higher range (>500 μmol/l) [49]. Smith et al. [50] also reported normal outcomes in 5 patients with untreated blood Phe levels between 360 and 600 μmol/l compared to matched controls. The number of patients having Phe levels just above 360 or just below 600 μmol/l was not reported. Because of limited data this publication was not considered [50]. An analytical shortcoming of previous studies is that patients were arbitrarily divided into subgroups. To examine the impact of Phe exposure in a vulnerable phase of brain development consider the use of more informative models like Widaman [51] did in maternal PKU. Therefore, we cannot give any definitive conclusions and consequently have decided to adopt a cautious approach. The evidence that supports treatment is of suboptimal quality. The evidence that supports no treatment is of better quality. However, the number of patients with blood Phe levels just below 600 μmol/l is considered too low and a different statistical analysis would be more informative. We recommend that patients with an untreated Phe concentration between 360 and 600 μmol/l should be treated during the first 12 years of age particularly as good metabolic control during childhood appears essential to prevent cognitive function impairment in PKU [52, 53].

For patients ≥12 years old with untreated Phe levels <600 μmol/l follow-up at a lower frequency is recommended, but remains particularly important in women due to the risks associated with maternal PKU when blood Phe levels are >360 μmol/l. Women need to be advised at each clinic that dietary treatment or BH4 therapy (or both) is essential pre-conception and during pregnancy. Some may consider that during child bearing years, women should continue a small dose of Phe-free L-amino acid supplements to help retain acceptance of its taste, but this practice remains unproven.
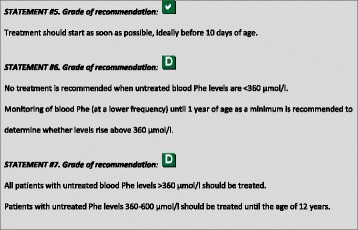


### Treatment for life

Since the introduction of NBS and early treatment, patients with PKU no longer develop profound and irreversible intellectual disability. Over the last 40 years, studies have demonstrated that it is unsafe to stop treatment during childhood and pre-adolescence [54, 55]. The foremost question now is if patients should be treated thoughout adulthood. There are no studies distinguishing the effect of Phe levels during different life phases (childhood, adolescence, adulthood). Also different terminology, target Phe levels and treatment strategies are given in published studies and consequently hamper a definitive conclusion. Here we describe studies in PKU patients who are continiously treated, on relaxed or discontinued diets and returned to diet.

Bosch et al. [56] reported that most early and continuously treated adults had a normal HRQoL even though dietary treatment is burdensome. Recently, a PKU related HRQoL questionnaire was developed, which assesses PKU-specific issues [57]. Bosch et al. [58] reported good HRQoL in 104 treated adult PKU patients with this PKU-specific and general questionnaire. Concerning neurological functioning, Fonnesbeck et al. [52] demonstrated an increased risk for low IQ with increasing Phe levels throughout life with a stronger association between blood Phe measured <6 years than later. In contrast, the meta-analysis of Albrecht et al. [59] indicated stable (but non-optimal) neurospychological speed test results with blood Phe levels between 750 and 1500 μmol/l. However there were too little data to exclude the possibility that lower Phe levels could improve performance [59]. Over a 5 year period in adulthood, Weglage et al. [60] reported that the IQ, information processing and attention of 57 early treated PKU (ETPKU) adult patients remained constant, despite elevated blood Phe levels [60].

In patients on a relaxed diet, Bik et al. [61] reported that HRQoL was good in some of the adults, whereas others suffered from severe emotional stress. In a German study, Simon et al. [62] described that a lower number of patients with PKU had stable relationships and patients reached independency at a later age compared with the general population. It is unclear how these adults were treated, but probably dietary treatment was relaxed as this is the usual practice in Germany.

Adults with PKU who discontinued the low-Phe diet during adolescence have been reported to show significantly slower reaction times [63] and subtle differences in inhibition, attention and working memory [64] compared with adults on dietary restrictions and control groups. The older group (>32 y) of Weglage et al. [60] performed slower in terms of information processing, which might be related to their early relaxation of diet. Dietary discontinuation during adolescence was concluded by Koch et al. [65] to be associated with poorer outcomes in adulthood regarding intellectual ability, achievement test scores and increased rates of medical and behavioural problems.

Some patients who experience suboptimal outcomes and return to diet improve. In adults, the reported neurological complications (*n* = 4) [66] and vision loss (*n* = 2) [67, 68] all improved or even reversed when Phe-restricted diet with Phe-free L-amino acid supplements was reinstituted [66–68]. In addition Schmidt et al. [69] reported reversible effects on sustained attention and calculation speed in a trial with 15 adults. Ten Hoedt et al. [70] showed in a randomized double-blind cross-over design study that short-term high Phe levels had a significant direct negative effect on mood and sustained attention in 9 adults. Returning to dietary restrictions has been shown to improve HRQoL in many of the adults with PKU who have been studied [61, 71]. However, it is possible that adults who have no desire to return to diet may not participate in studies.

Overall it is unclear how many adults experience suboptimal outcomes that have impact on daily functioning. It is also not fully understood which consequences during adulthood are due to Phe levels before adulthood and/or during adulthood, and which of these consequences is improved by decreasing blood Phe during adulthood. Neither, it is clear if Phe levels during adulthood will impact outcome in elderly patients.

As there is currently no strong evidence that it is safe to discontinue dietary treatment in adults, treatment for life is recommended, even though it is acknowledged that dietary management is associated with significant patient burden. Returning to the diet is very challenging if patients have eaten high protein foods and/or find the Phe-free-L-amino acid supplements distasteful. Patient motivation should be strong with a supportive family network and metabolic team to overcome any barriers.
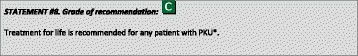


*Patients ≥12 years with untreated Phe levels <600 μmol/l do not require treatment (statement #7).

### Life-long follow up

Evidence from a systematic review demonstrates that significant sub-optimal outcomes exist in ETPKU adults. Issues include EF deficits, attention problems, decreased verbal memory, expressive naming and verbal fluency, as well as social and emotional difficulties [5]. ETPKU adults usually show a clear relationship between concurrent blood Phe concentrations and certain aspects of brain function, brain metabolism and differences in myelination as summarized by van Spronsen et al. [72]. Some adults who have not been treated early and continuously have been reported to develop neurological complications such as leukoencephalopathy, spastic paraparesis, brisk reflexes, tremor, Parkinsonism, psychiatric symptoms (*n* = 4) [66] and vision loss (*n* = 2) [67, 68]. Tremors have also been detected in ETPKU, although they are more frequent and severe in late treated patients [73]. At present, it is not known how many patients have neurological and psychological problems and which adult PKU patients have a higher risk of these problems. Many adults with PKU have a vegan-like diet but may not take Phe-free L-amino acid supplements [74] and consequently may be at risk of micronutrient deficiencies [75]. There is increasing reports of females (and not males) with PKU being overweight and obese [76, 77]. The risk of comorbidities makes dietary management more complex [78]. The risk of low bone density has widely been acknowledged but the risk of bone fractures is still unclear [79].

In PKU, life-long, systematic follow-up is recommended independent of the degree of adherence and (non-) treatment choice, to screen for long-term complications at any life stage, and provide appropriate support to patients. In addition, it is not known if there will be further complications when adult PKU patients advance in age, such as neurodegeneration or movement problems. By collecting data, we should be able to identify if patients are likely to deteriorate and which patients are at special risk of deterioration and why.
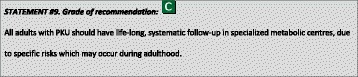


## Treatment goals and follow-up

The primary goal of treatment is normal neurocognitive and psychosocial functioning. Blood Phe concentrations remain the best surrogate measure, and should be monitored regularly, aiming for blood Phe levels that stay within a given target treatment range, defined for a given age. Discussions on target ranges have focused primarily on the upper blood Phe level but there is little data to support the lower target level. The widely used lower target level of 120 μmol/l is derived from published cases describing adverse consequences at very low Phe levels [80, 81], and from past knowledge that the primary use of the Guthrie test was not sensitive in detecting lower Phe levels. It is now well established that blood Phe decreases during the day with the highest blood Phe attained early in the morning, following an overnight fast [82]. We advise a lower target level at 120 μmol/l until more data is available.

When trying to reach consensus about the upper target Phe concentration for treatment in PKU, comparison of studies was hampered by various factors:

- Studies report blood Phe in different ways (e.g. concurrent, lifetime as a mean, lifetime as median, or lifetime means of medians). Studies use different methods to measure blood Phe (past data were sometimes based on the semi-quantitative Guthrie or more reliable fluorometric enzyme analysis but more recently amino acid concentrations were usually measured by high-performance liquid chromatography and tandem mass spectrometry that are more precise). Differences between the methods (except for Guthrie method) are relatively small [83–85].
- Studies use different Phe samples such as venous serum, venous plasma and DBS. Past studies are largely based on plasma Phe levels, where it is now routine practice to perform DBS measurements. Differences between venous serum and venous plasma are usually regarded as minimal with a variation of 1% [86], but differences between DBS and plasma may be greater with DBS being reported to be 8–26% lower [84, 86, 87]. It should be considered that a higher plasma Phe is likely to result in a higher variation between DBS and plasma.
- There can be variations in Phe results due to variety in measurement in the DBS itself, haematocrit, the volume taken from the DBS, and the punch location [87–90]. At the same time, it is also reported that reliable Phe levels can be estimated within a minimum size of blood spot [91].
- Studies do not consistently include confounding factors such as maternal education, socioeconomic status and age at start of treatment.

The statements in this guideline recommend blood Phe as upper target levels where reported studies used means or mean of medians. Therefore, these upper target levels are probably on the safe side (considering current evidence), so even with differences in blood levels due to sample type, we still consider we have a reasonable upper target for Phe levels.

### Target Phe levels for children and adolescents

Albrecht et al. [59] performed a meta-analysis including 20 studies focusing on neuropsychological speed tests of 7 different categories. In total, 509 patients (229 children, 106 adolescents and 174 adults) and 433 controls participated in these studies. The meta-analysis predicted no differences with controls when concurrent Phe concentrations reached 320 μmol/L for children between 7 and 13 years and up to 570 μmol/L for adolescents between 13 and 18 years of age [59]. Waisbren et al. [53] performed a meta-analysis examining the correlation between IQ and Phe levels reported in 40 different publications. They concluded that a difference in Phe level of 100 μmol/l between birth to 6–12 years predicted a difference in IQ between 1.3 to 3.1 points in patients whose Phe levels ranged from 423 to 750 μmol/l. With lifetime Phe levels, an increase of 100 μmol/l predicted an average 1.9 to 4.1 point reduction in IQ over a range of Phe from 394 to 666 μmol/l [53]. For example, someone with a Phe level of 500 μmol/l, on average had a 1.9 to 4.1-point lower score on an IQ-test compared to someone with a Phe-level of 400 μmol/l. Fonnesbeck et al. [52] performed a meta-analysis of 17 studies (432 individuals with PKU, aged 2–32 years) and addressed the relationship between the probability of an IQ less than 85 and Phe levels. Both life time Phe levels (more than 12 months before IQ measurement) and concurrent Phe levels (within 6 weeks of IQ-measurement) were considered [52]. The healthy population probability of an IQ less than 85 was approximately 15%. For PKU patients the probability was 14% when the mean Phe level during the time frame of ≥6 years of age was 400 μmol/l but increased to 20% when the mean Phe level was 600 μmol/l. Before <6 years of age the probability was already 19% when the mean Phe level was 400 μmol/l and increased to 30% when the mean Phe level was 600 μmol/l. A stronger association was observed between Phe levels during early childhood and later IQ. There was no strong association between concurrent Phe levels and IQ [52]. Taken together, in childhood, the meta-analyses of Albrecht et al. [59] and Waisbren et al. [53] suggests an upper target Phe concentration of 320 (age 7–13 years), and 423 μmol/L (birth to 6/12 years), while the meta-analysis of Fonnesbeck et al. [52] suggested that a mean of 400 μmol/L (<6 years) is already too high as it was associated with an increased risk of an IQ <85. It should be noted that the primary papers considered in these meta-analyses are mostly non-experimental designs such as (historical) cohorts, cross-sectional designs and case series, which in turn decreased the quality of these analyses.

Diamond et al. [48] showed in 37 PKU patients aged 6 months to 7 years that those with concurrent Phe levels (mean Phe from a 6 week period preceding testing) of 360–600 μmol/l performed less well in EF tasks requiring working memory and inhibitory abilities than did children with concurrent Phe levels <360 μmol and controls. In addition, PKU children with concurrent Phe levels 360–600 μmol/l had significantly lower IQ scores than did control subjects, although all participants scored within the normal range [48]. In a study by Leuzzi et al. [92], 9 PKU patients with Phe levels >400 μmol/l performed worse than 5 PKU patients with levels <400 μmol/l and IQ- and age-matched controls (8–13 years) in all 7 tests, although not all differences were significant. PKU patients with Phe levels <400 μmol/l performed comparably with controls in all tests but the Elithorn’s Perceptual Maze Test [92]. In addition, Huijbregts et al. [93] found that 38 PKU patients with concurrent Phe >360 μmol/l performed significantly worse in several tests targeting EF than matched controls. Patients with concurrent Phe levels <360 μmol/l (*n* = 29) did not differ from controls and performed significantly better than patients with concurrent Phe levels >360 μmol/l [93].

Schmidt et al. [69] (included in the meta-analysis of Albrecht et al. [59]) reported 4 groups of PKU patients (mean age 9 years). Group A had good metabolic control (from birth to the age of 9 years) and had a concurrent Phe level of 240 μmol/l (*n* = 31). Group B had good metabolic control up to the age of 9 years, but had a concurrent Phe level of 620 μmol/l (*n* = 30). Group C and D were not in good metabolic control and had a concurrent Phe level of 520 μmol/l and 970 μmol/l (*n* = 32). Group A performed as well as the control group and better than group B, C and D for sustained attention and calculation speed tests. All the other groups performed worse than the control group [69].

Jahja et al. [94] examined inhibitory control, cognitive flexibility and motor control in 3 groups of PKU patients (aged 6–15) with different lifetime Phe levels and healthy controls (*n* = 73). The 3 groups had lifetime Phe levels of ≤240 μmol/L (*n* = 10), between 240 and 360 μmol/L (*n* = 33) and ≥360 μmol/l (*n* = 21). The patients with Phe levels below ≤240 μmol/l performed better than the other 2 PKU groups and equally well as the control group [94]. However, despite statistical significant differences, this was not considered clinically significant.

Moyle et al. [95] performed a meta-analysis of neuropsychological testing. PKU literature often combines data from children, adolescents and adults but this compromises the ability to interpret the results. Moyle included 11 papers focusing on adolescents (13–18 years) and adults (>18 years). The level of dietary adherence was not uniform, although the majority of patients was following a relaxed diet at the time of testing. Additionally, the matching criteria and type of control groups differed across studies. The results from the study indicated that continuously treated PKU patients (without correcting for treatment adherence), while displaying no significant weakness in working memory, are likely to show reduced levels of functioning across a range of different cognitive functions (IQ, attention, inhibition, processing speed, and motor control) compared to controls [95].

Weglage et al. [60] examined adults with early-treated classical PKU to assess neurological and neuropsychological performance. At baseline, 28 patients were aged <32 years and 29 were >32 years. The older group relaxed the diet at the age of 10 years, while the younger group relaxed the diet in early adulthood. Significant differences were observed in Phe levels between the ages of 11 and 16 years. When studied for a 5 year period in adulthood, both groups remained constant in their performance. The older group, however, performed more slowly in testing for information processing, which might be related to their early relaxation of diet. From the age of 11 until 16 years, in the younger age group the median annual Phe varied between 496 and 707 μmol/l and for the older group, between 750 and 1038 μmol/l [60]. In summary, for adolescents the meta-analysis of Albrecht et al. [59] recommended a target Phe level of 570 μmol/l (age 13–18 years), whereas the findings of Weglage et al. [60] suggest an upper target level between 496 and 707 μmol/l (age 11–16 years). The meta-analysis of Fonnesbeck et al. [52] and Waisbren et al. [53] are more difficult to interpret as they refer to lifetime Phe levels.

The evidence for patients <12 years of age is strong indicating that a Phe concentration of 360 μmol/l should be considered as the upper target Phe concentration. It could be argued that within this age group the upper target Phe levels needs to be lower (Schmidt et al. [69], Jahja et al. [94]), but at present time the evidence to lower the upper target Phe is not robust enough. If possible, meta-analysis of the data available studying the relationship between neurocognitive and neuropsychological outcome and blood Phe concentrations, examining if upper Phe levels other than 360 μmol/l give even better results are necessary, stressing the need for collaboration on an international level [51].

The evidence for patients >12 years of age is mainly indirect, as there are no studies investigating the effect of Phe levels during adolescence in patients who were in good metabolic control during childhood. Taking into acount the lower grade of evidence, an upper target Phe level at 600 μmol/l between ages 12 and 18 years is recommended.

### Target Phe levels during adulthood

In adulthood the goal of treatment is to achieve normal neurocognitive and psychosocial functioning. As previously discussed, it is not fully understood which PKU adult outcomes are associated with increased Phe levels during adulthood and there are no large controlled longitudinal studies to help determine the optimal upper target blood Phe levels. Further data collection by long-term international collaborative studies is required to help direct current recommendations.

In the double-blind randomised placebo-controlled cross-over trial of Ten Hoedt et al. [70], 9 patients received Phe-loading and placebo-Phe-loading. Mean plasma Phe concentrations were 1259 μmol/L (±332 μmol/l) versus 709 μmol/l (±322 μmol/l), respectively. The higher Phe levels significantly worsened mood and sustained attention [70]. In Schmidt’s et al. (1996) controlled experimental study, 15 early treated adults with normal IQ were tested 3 times; with their usual diet, a Phe-restricted diet and again their usual diet. Mean Phe levels were 1320 μmol/l (720–1800 μmol/l), 630 μmol/l (280–966 μmol/l) and 1410 μmol/l (1040–2200 μmol/l), respectively. Sustained attention and calculation speed improved significantly with the lower Phe levels [69].

Channon et al. [64] compared 25 treated adults on diet with 25 adults who stopped treatment from 10 years of age onwards. The treated adult patients had a better performance for IQ, n-back accuracy and flanker speed, although the Phe levels differed significantly from 5 years of age onwards between the 2 groups. The range of mean 4-yearly Phe levels was 460–870 μmol/l for the adults who remained on treatment, and 560–1410 μmol/l for the off-diet group. The on-diet adults performed worse compared to controls regarding n-back speed [64]. With these studies, it is difficult to interpret if consequences are due to Phe levels during childhood, adolescence or adulthood. Adulthood enables more invasive techniques to be used to determine safe Phe concentrations. Hoeksma et al. [96] using positron emission tomography, showed that plasma Phe concentrations >600–800 μmol/l decreased cerebral protein synthesis rates in adults (*n* = 16) [96]. In several studies in PKU, but mainly with adolescents and adults, no white matter alteration (WMA) is observed when blood Phe is <300 μmol/l or in some cases <600 μmol/l [49, 97–100]. Blood Phe control and its impact on oxidative stress has also been considered. Oxidative stress occurs in neurodegenerative disease and the brain has relatively low levels of antioxidant defences. Sanayama et al. [101] reported oxidative stress changed greatly at a blood Phe level of 700–800 μmol/l (*n* = 40) and thereby recommended Phe levels <700–800 μmol/l [101].

The evidence, as strong or weak as it is, indicates 600 μmol/l as the upper target level, while no study could be found to support an upper target blood Phe level of 360 μmol/ [102]. It is recognized that an upper target Phe level of 600 μmol/L increases the dietary burden of care and may provide more challenges for patients returning to dietary treatment but this was not a determining factor in recommending this upper target Phe level.
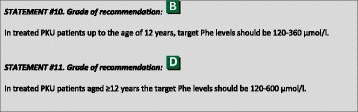


(See subparagraph maternal PKU for recommendations regarding maternal PKU) 

### Biochemical marker used for assessment of metabolic control

Blood Phe levels (but not Phe fluctuations and Phe: Tyr ratios) are the primary reported markers of metabolic control [52, 53, 59]. Treatment is adjusted according to the blood Phe level. The effect of a single Phe levels outside the target range is not easily measured. Phe fluctuations over 24 h appear to be more related to uneven administration of Phe-free L-amino acid supplements [103], rather than the fasting/postprandial state or uneven distribution of natural protein allowance [82, 104].

There are data indicating that fluctuations in Phe (often measured as SD or SEE) can be a predictor of IQ [105–107], EF [106] and motor control [94], although the literature is inconsistent [108]. As Cleary et al. [109] described, it is difficult to distinguish the effect of more severe PKU and/or poor metabolic control from the effects of Phe fluctuations. Additionally, further research is needed to examine the differences between the short-term and long-term effect of Phe fluctuations [109].

Considering the Phe: Tyr ratio, it is hypothesed that an increased Phe: Tyr ratio leads to dopamine deficiency as Phe and Tyr compete to cross the blood–brain barrier [48]. Jahja et al. [94] concluded, using multiple regression analysis (*n* = 64), that increased Phe: Tyr ratios were associated with poorer inhibition control [94]. Sharman et al. associated Phe: Tyr ratios with EF (T-scores from Behaviour Rating Inventory of Executive Function) in 2 papers partially using the same subject sample (*n* = 11 and *n* = 12). They suggested that a lifetime Phe: Tyr ratio of <6 was associated with a normal EF outcome, but this requires further evaluation by others [110, 111]. Furthermore, in 2012, Sharman et al. found significant correlations between depressive symptoms and long-term exposure to either a high Phe:Tyr ratio or low Tyr, although the 18 adolescents with PKU scored within the normal range for depressive symptoms [112]. Luciana et al. [113] reported an association of the Phe: Tyr ratio with several aspects of cognitive functioning in a group of 18 PKU patients. Again, it was difficult to distinguish between the effect of Phe: Tyr ratio and the elevated Phe levels. Probably, the Phe: Tyr ratio is useful, but as the Tyr concentration depends on the timing of blood sampling [82, 114], the marker is only of value if measured after an overnight fast. Therefore, the exact value of the Phe: Tyr ratio in addition to blood Phe measurements remains to be determined.
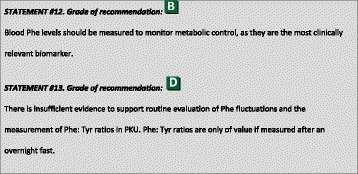


### Frequency of blood Phe measurements and outpatient visits

Patients are monitored with home blood sampling and outpatient visits. The effect of frequency of contact or regularity of blood sampling on adherence has not been adequately assessed in PKU. Frequent contact during the first year of life is essential to instruct parents and help attain good metabolic control. Regular follow-up during adolescence is also crucial as it is well established that blood Phe control deteriorates [115]. After the age of 12 years, patients with PKU should aim for blood Phe levels of 120–600 μmol/L. It is essential that adolescents are supported throughout the transition process until they are established and confident in an adult care environment; they should be encouraged to take responsibility for self care, taking regular blood Phe samples, attending age appropriate outpatient clinics with suitable education programmes.

We suggested the following minimum frequencies of blood sampling and minimum outpatient visits for each age group:
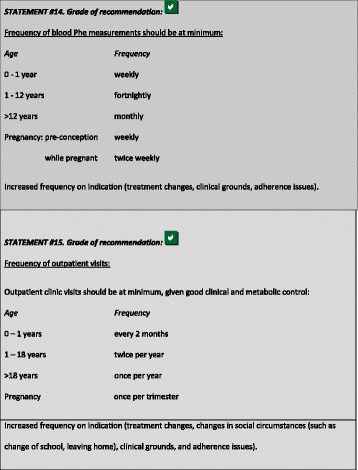


During the first year of life and throughout pre-conception and pregnancy, weekly (telephone) contact with health professionals is important to provide close support to patients and their families. Various life events, such as change of school, starting employment, living independently, as well as adherence issues (e.g. during adolescence) may necessitate a higher frequency of blood Phe testing and/or visits.

It is important that blood Phe samples should be obtained at the same time of the day. To estimate the highest Phe value of the day and reliable Tyr levels, blood samples should be collected in the morning after fasting overnight. Blood Tyr levels taken at different times may be increased by the tyrosine intake from Phe-free L-amino acid supplements.

The time between bloods sampling and patients/parents receiving the results should be minimized, aiming for less than 5 days. In special situations such as infancy and maternal PKU, results should be available within 2–3 days of blood sampling. This requires home monitoring systems instead of home sampling.

At each outpatient visit, the following should be conducted: a medical and dietary history, assessment of anthropometry including body mass index estimation, and a physical and neurological examination, especially observing for clinical signs of Phe toxicity and nutrient (including Phe) deficiency [80, 81]. Clinic reviews should always include a discussion on treatment issues and mental and physical health (e.g. neurological and psychiatric issues, behaviour and mood). Any additional investigations necessary are outlined in Table 2.
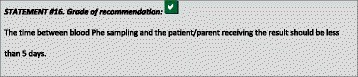

**Table 2:** Minimum requirements for the management and follow-up of patients with PKU

	Childhood (<12 y)	Adolescence (12–18 y)	Adulthood (≥18 y) excluding maternal PKU	Maternal PKU
Outpatient visit	Given good clinical and metabolic control: Age 0–1 years: every 2 months Age 1–12 years: twice per year Extra clinic visit as indicated	Given good clinical and metabolic control: twice per year Extra clinic visit as indicated	Given good clinical and metabolic control: once per year Extra clinic visit as indicated	Given good clinical and metabolic control: once per trimester Extra clinic visit as indicated
Clinical nutritional assessment	Every outpatient visit: dietary assessment (3-day food record/24 h recall), anthropometric parameters (weight, height, BMI) and clinical features of micronutrient and Phe deficiency (especially anorexia, listlessness, alopecia, perineal rash)	Every outpatient visit: dietary assessment (3-day food record/24 h recall), anthropometric parameters (weight, height, BMI) and clinical features of micronutrient and Phe deficiency	Every 12–24 months: dietary assessment (3-day food record/24 h recall), anthropometric parameters (weight, height, BMI) and clinical features of micronutrient and Phe deficiency	Every outpatient visit:dietary assessment (3-day food record/24 h recall) and weight
Metabolic control	Age 0–1 year weekly Phe Age 1–12 years fortnightly Phe Increased frequency as indicated Annually: plasma amino acids	Monthly Phe Increased frequency as indicated Annually: plasma amino acids	Monthly Phe Increased frequency as indicated Annually: plasma amino acids	Pre-conceptionally: weekly Pregnancy: twice weekly Increased frequency as indicated Pre-conceptionally: plasma amino acids
Biochemical nutritional assessment	Annual measurement of plasma homocysteine and/or methylmalonic acid, haemoglobin, MCV and ferritin. All other micronutrients (vitamins and minerals including calcium, zinc, selenium) or hormones (parathyroid hormone) if clinically indicated			Pre-conception and at the start of pregnancy: folic acid, vitamin B12, plasma homocysteine and/or methylmalonic acid, ferritin, full blood count Pregnancy: when indicated
Bone Density	BMD measurement only indicated when there are specific clinical reasons or when patients are known to be at particular risk of metabolic bone disease	The first measurement of BMD should be undertaken during late adolescence - When BMD is abnormal, DXA (with or without change of treatment) should be repeated after 1 year. If osteoporosis (BMD < -2.5 SD) persists despite optimization of diet and physical activity, other possible causes of osteoporosis should be investigated. Treatment (including consideration of bisphosphonates) should be determined by osteoporosis severity. - If BMD results are still low but stable, yearly measurement is unnecessary. - When BMD is normal, no repeat measurement is necessary. Further study need only be considered when there are clinical reasons to do so.	BMD measurement is only indicated when there are specific clinical reasons or when patients are known to be at particular risk of metabolic bone disease	Not indicated
Neurocognitive functions	Only neurocognitive tests when indicated.	Testing at age 12 years Proposed domains of neurocognitive testing: IQ, perception/visuospatial functioning, EF (divided into inhibitory control, working memory and cognitive flexibility) and motor control. Extra neurocognitive tests as indicated.	Testing at age 18 years Proposed domains of neurocognitive testing: IQ, perception/visuospatial functioning, EF (divided into inhibitory control, working memory and cognitive flexibility) and motor control. Extra neurocognitive tests as indicated.	Not indicated
Adaptive issues (e.g. clinical relevant behavioural problems)	Annually: clinical assessment/discussion	Annually: clinical assessment/discussion Screening at age 12 years	Annually: clinical assessment/discussion Screening at age 18 years	Not indicated
Neurological complications	If neurodegeneration occurs	If neurodegeneration occurs	Annually: clinical examination	Not indicated
Psychosocial functioning and wellbeing and QOL	Annually: Clinical assessment/discussion Once during childhood: (PKU-)QOL questionnaire	Annually: Clinical assessment/discussion Once during adolescence: (PKU-)QOL questionnaire	Annually: Clinical assessment/discussion Once during adulthood: (PKU-)QOL questionnaire	Especially in case of not becoming pregnant, the patient may need support
Psychiatric examination	At onset of symptoms of psychiatric disturbances	At onset of symptoms of psychiatric disturbances	At onset of symptoms of psychiatric disturbances	Not indicated
White matter abnormalities (MRI)	When there is an unexpected clinical course and/or unexpected neurological deficits	When there is an unexpected clinical course and/or unexpected neurological deficits	When there is an unexpected clinical course and/or unexpected neurological deficits	Not indicated
Age group specific investigations	/	/	/	Ultrasound at 18–22 weeks of pregnancy with screening for organ development (especially if there is lack of optimal metabolic control)
				Echocardiogram in all infants who are conceived by women with either high blood Phe levels or poor maternal blood Phe control during pregnancy

### Metabolic team and transition

All patients should be treated in a specialized metabolic centre with a specialized metabolic laboratory. The minimum health professionals within a team for patients of all ages should be a metabolic physician and a dietician with experience in IMD. Access to a psychologist is requested by the ESPKU patient organization [11] while we strongly advise access to a (neuro)psychologist and social worker. It is recognized that PKU is a IMD possibly necessitating may necessitate the support of professionals outside the core team. That support can be for financial issues and beyond. Although in many countries adult patients are followed up by a paediatric team [116], it is important that metabolic teams prioritise the establishment of an adult metabolic service, lead by an adult metabolic physician, specifically trained in the management of IMD.

The process of transferring children to adult care should be conducted under a carefully structured ‘transitional’ process, beginning from around the age of 12 years. During this time, management should change from being parent/caregiver directed to patient controlled. This latter process must occur even if the patient is staying under the same paediatric service. Patients and families need an individualized care plan and timetable for transition, together with detailed information about the adult centre. This should be jointly written with teenagers, caregivers, and health professionals. This plan should include treatment goals, a timetable for transfer, and ensure there is a consistent approach between all health professionals. It should also provide a mutual understanding of the transition process. It has been demonstrated in PKU, with careful planning, close liaison between paediatric and adult teams, and patient and caregiver involvement, that most patients are able to make a successful transition to adult care [117]. There is no right time or age for the subsequent transfer of patient care to the adult treatment centre to occur but is commonly between 16 to 18 years of age, although some flexibility may be required depending on the maturity and circumstances of the patient.
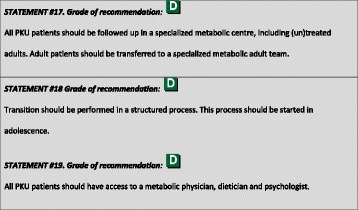


### Nutritional follow-up

The nutritional status of patients varies according to PKU severity and type of treatment. Except for patients on a normal diet (MHP and fully BH4-responsive patients), the majority follow a low natural protein diet with limited or no animal protein sources. The major source of micro-nutrients is from supplemented Phe-free L-amino acids and if the intake of Phe-free L-amino acid supplements is sub-optimal, this will increase the risk of micronutrient deficiency (e.g. iron, zinc, selenium and vitamin B12) [118–120].

Clinical symptoms of nutrient deficiency are rarely reported, and are mainly described for vitamin B12 deficiency in patients who have reduced or stopped their micronutrient supplement or Phe-free L-amino acid supplements while following a vegan-style diet [121, 122]. For some nutrients, the bioavailability appears sub-optimal (e.g. zinc [118, 123, 124] and iron [118, 125–127]).

Functional markers of micronutrient status (ferritin, hemoglobin, MCV for iron; methylmalonic acid and total homocysteine in serum for vitamin B12) are useful to detect iron and vitamin B12 deficiency as their plasma concentrations are not fully related to their nutritional status (e.g. functional vitamin B12 deficiency) [128, 129].

In addition, some studies have demonstrated high folate levels in patients associated with the high folate content of Phe-free L-amino acid supplements with added vitamins and minerals [118, 127, 130]. The long- term consequences of folate overload in PKU have not been assessed. Deficiencies of other micronutrients are rarely reported.

The nutritional follow-up requires the monitoring of anthropometry, body mass index (BMI), clinical signs of nutrient deficiency, nutrient intake and biological biomarkers to detect subclinical micronutrients excess or deficiencies. Accessing information about frequency and amount of L-amino acid supplements prescribed and delivered to a patient’s home will give some indication about patient adherence, but still does not guarantee that products are consumed.
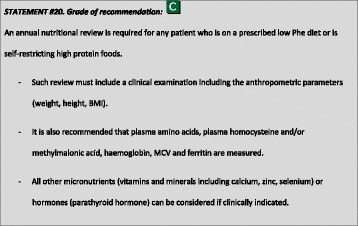


### Bone density

The main factors influencing bone density are calcium and vitamin D status, the quality of bone proteins, physical activity, endocrine status, genetic and environmental factors.

#### Osteopenia and PKU

Osteopenia and osteoporosis in PKU has been described for many years. Definitions of osteopenia and osteoporosis are highly heterogeneous between studies and do not align with World Health Organization (WHO) standards and the International Society for Clinical Densitometry (ISCD) positions on bone mineral density (BMD) measurement [79]. According to the ISCD, fracture history must be assessed alongside BMD Z-score before diagnosis can be made [131]. In adult patients, WHO guidelines require T-scores to diagnose osteopenia (T-score between −1 and −2.5) or osteoporosis (T-score below −2.5) [132]. In all patients Z-scores can be used, except in males older than 50 years and postmenopausal women in which the use of T-scores is advised.

There have been 3 systematic reviews on bone density in PKU: Enns et al. [5] (9 papers published after the year 2000), Hansen et al. [133] (16 papers) and Demirdas et al. [79] (13 papers) [5, 79, 133]. Enns et al. [5] found a sub-optimal outcome for bone health in PKU in all 9 studies. Hansen et al. [133], on a meta-analysis of 3 papers, showed a significantly lower spine BMD (0.100 g/cm^2^ ) in 67 subjects with PKU compared to 161 controls. These papers included early and late treated PKU patients; only 1 corrected for reduced height. Demirdas et al. [79] performed a meta-analysis with only ETPKU patients. Mean total body (3 studies; *n* = 133), lumbar spine (7 studies; *n* = 247), and femoral hip (2 studies; *n* = 78) BMD Z-scores in patients with PKU were lower than in their healthy peers, but well within the normal reference range, respectively −0.45 (95% CI −0.61, −0.28); −0.70 (95% CI −0.82, −0.57); −0.96 (95% CI −1.42, −0.49) [79].

#### Fracture risk in PKU

An increased fracture risk has been infrequently described [134].

#### Pathophysiology of osteopenia in PKU

##### Nutritional factors and osteopenia in PKU

Earlier studies described calcium and vitamin D deficiencies [135, 136], but the calcium and vitamin D content of current Phe-free L-amino acid supplements exceed requirements. Pérez-Dueñas et al. [73] showed a positive correlation between BMD and mineral intake and concluded that the correct intake of Phe-free L-amino acid supplement was necessary for bone mineralization. They found, however, that vitamin D supplements improved BMD in a cohort of patients with inadequate intake (*n* = 6/28) [135]. Despite adequate calcium and vitamin D content of Phe-free L-amino acid supplements, osteopenia is still identified in patients on strict low Phe diet and good metabolic control [137]. Patients with PKU also have an increase of calciuria, demonstrating no calcium deficiency [138]. Therefore, micronutrient intake is not the only causative factor of bone disease in PKU. Interestingly, docosahexaenoic acid (DHA) deficiency has also been associated with osteopenia in PKU [137].

##### The severity of PKU

Osteopenia has not been observed in untreated MHP [5, 131, 139, 140]. It has been described in classical PKU with various calcium metabolism profiles. In patients with classical PKU and poor diet, osteopenia was associated with an increase in parathyroid hormone (PTH) and alkaline phosphatase activity, both of which are related to calcium or vitamin D deficiency [141, 142]. However, even classical PKU patients on strict diet with normal alkaline phosphatase and PTH activities may have osteopenia associated with osteoporosis pathophysiology.

##### Alteration of bone metabolism

The metabolic profile of calcium metabolism in PKU patients is identical to that observed in classical osteoporosis (normal blood calcium, phosphorus, alkaline phosphatase and PTH associated with an increase of calciuria and C-terminal telopeptide). Demirdas et al. [79] reported that bone turnover results were ambiguous and that it is not clear from studies whether bone formation is decreased or bone resorption is increased. This may be partly due to heterogeneity in both markers and populations with regard to age [79].

##### Natural protein intake

Bone health also depends on the quality of its protein structure as evident by the bone fragility observed in osteogenesis imperfecta. The impact of overall protein status, including the biological value of intact protein versus Phe-free L-amino acid supplements and the percentage of protein derived from natural protein, is often not considered in studies [79]. Miras et al. [137] described 43 patients with classical PKU on a strict low-Phe diet, 14% of whom had mineral bone disease (MBD). The main difference between the group with and without MBD was the natural protein intake (14.33 +/− 8.95 g/day in the group with MBD vs. 21.25 +/− 20.85 in the group without MBD) [137]. Solverson et al. [143] showed an improvement of bone density in a group of mice treated with a low-Phe glycomacropeptide compared with Phe-free L-amino acid supplement [143], and Miras et al. [137] identified an absence of bone disease in 12/12 PKU patients treated by BH4, which allowed a higher natural protein intake [137].

Practically an adequate intake of calcium and vitamin D, regular exercise and optimization of natural protein intake must be ensured. We suggest follow-up of BMD during late adolescence (statement #22), although there is no sound research data suggesting follow-up by DXA or other methods.
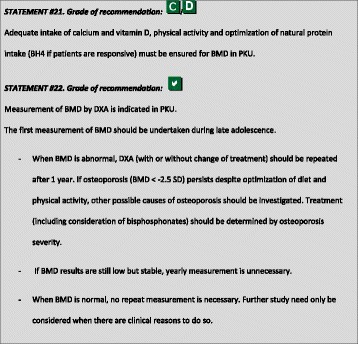


### Brain magnetic resonance imaging

PKU is associated with the occurrence of white matter (WM) abnormalities (WMA) on magnetic resonance imaging (MRI) in both early and late treated patients [144–146]. The pattern of WM involvement in ETPKU is characterized by patchy or diffuse symmetrical lesions of deep and periventricular WM (occipito-parietal, frontal, temporal) appearing as signal hyper intensity on T2-weighted and FLAIR sequences and, in a minority of subjects, as signal hypo intensity in T1-weighted sequences. Several controlled cross-sectional studies (with 21 to 77 PKU patients per study) showed that the extent and severity of WMA appear to be moderated by patient age and/or dietary adherence (as reflected by blood Phe levels), with older age and/or higher Phe levels associated with increased white matter involvement [147–153].

Whether these lesions have any clinical impact is unclear and the mechanisms involved in their pathogenesis are not known. No WMA in subjects with blood Phe levels <300–600 μmol/l have been reported [49, 97–100]. Weglage et al. [49] found no WMA in 31 PKU adolescent and adult patients with untreated Phe levels <600 μmol/l [49]. Bick et al. [97], Kono et al. [98], Lou et al. [99] and Manara et al. [100] found no WMA in respectively 2 PKU adults with (lifetime) Phe <360 μmol/l [97], 7 PKU children and adolescents with (concurrent) Phe <68–514 μmol/l [98], 2 PKU adolescents with Phe 200–300 μmol/l during childhood [99], 8 PKU adolescents with concurrent Phe <400 μmol/l or mean Phe-year <460 μmol/l [100].

However, other factors are involved as suggested by the occurrence of WM variation (improvement or worsening) in patients who did not change their Phe values, and the wide variability of WM involvement under similar value of blood and brain Phe [149, 150].

WMA are reported to be reversible. Two controlled studies [154, 155] showed an improvement of WMA (3 to 6 months) after lowering of blood Phe levels. Cleary et al. [154] reported improvement was primarily in those with reduced Phe levels <900 μmol/l and scans improved in all 5 patients with reduced Phe levels <400 μmol/l. White et al. [155] also found improvements in 12 PKU patients lowering their Phe levels from a mean of 653 (322) μmol/l to 409 (256) μmol/l [155]. Similar results come from single cases and small cohort studies [97, 156, 157].

Current neuroimaging techniques are not useful in monitoring the clinical outcome for ETPKU patients. Neuroimaging examinations should be reserved for those patients presenting with an atypical clinical course and/or unexpected neurological deficits or for research purposes.
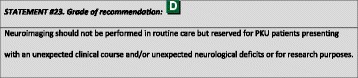


### Neurocognitive functioning

PKU patients have an increased risk of developing neurocognitive problems [52, 53, 95]. Gassio et al. [158] demonstrated more school problems in ETPKU than control subjects, probably related to the disturbed cognitive functions observed. Although the majority of ETPKU individuals have educational and professional achievements similar to their non-PKU siblings, they have more pronounced problems in social functioning and emotional wellbeing [70, 159]. The clinical relevance and the relationship to metabolic control need to be established in future research. Routine neurocognitive evaluations should be performed at 12 and 18 years of age in all patients. This corresponds with changes in treatment targets for blood Phe or life changes (e.g. change of school, living situation, job, transfer to adult clinic.). This recommendation will provide baseline data about neurocognitive functioning prior to any relaxation of blood Phe levels at the age of 12 years or at the time patients are starting their adult life. Referral to a (neuro) psychologist is strongly recommended if risk factors apply as stated in statement #24.

Supplying treating centers with the correct PKU profile for testing neurocognitive capacities in PKU patients for routine care at 12 and 18 years is still a challenge. In short, there are no PKU specific tests available to measure neurocognitive functions. While which test to use is largely a professional’s choice and/or centre dependent, the target should be a comprehensive neuropsychological assessment exploring cognitive performance across different domains. Reminding this, literature shows many aspects of neurocognitive functioning in PKU patients for which, at some point, phenylalanine-related impairments have been shown. These include perceptual skills, visuospatial abilities, and fine motor control (for an overview, see Janzen and Nguyen [160]). Whereas for these aspects of cognition, impairments were shown relatively consistently, there are other domains such as language, verbal fluency, and long-term memory for which impairments were shown incidentally. The most consistent phenylalanine-related impairments have been observed in the domain of executive functioning (EF, e.g. inhibitory control, working memory, cognitive flexibility) (for an overview, see Christ et al. [161]). The level of complexity of the tasks that were used in neuropsychological assessments (in other words: the level of executive control that was required) seems to be a determining factor in whether or not impairments will be observed. Therefore, it is important that any form of neuropsychological assessment that will be chosen for the monitoring of PKU-patients captures these different levels of complexity. In young and young adult PKUs the assessment of some complex cortical functions, such as EFs (reasoning, planning, flexibility, and monitoring), visuo-motor coordination and speed of processing may be a sensitive tool in detecting possible neuropsychological impairment. However, there are some issues to be solved in research setting before we can introduce EF in routine clinical practice 1) the consistency of EF alterations in serial evaluations across different ages; 2) the predictive value of EF alterations with respect to later neurocognitive functioning and real life adaptation. Knowing all these issues and the time consuming aspects for staff members of these test, it will be important to continue to study links with instruments that have already shown to be more or less sensitive in picking up phenylalanine-related impairments in PKU [162].
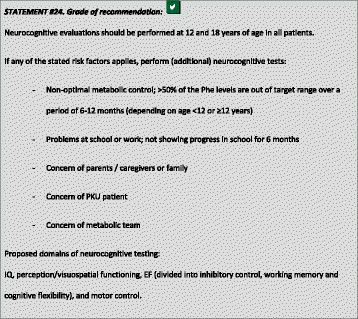


### Psychosocial functioning

Studies evaluating the HRQoL of patients with ETPKU demonstrated a normal HRQoL compared to the general population [56, 62, 163, 164], with the exception of 1 study reporting a lower score on the cognitive domain in adults [165], and 1 study demonstrating a lower HRQoL in a group of Italian children [166]. This contrasts with the view of patients and professionals who experience or observe stress associated with the burden of the diet. Normal HRQoL results may be due to the use of generic questionnaires or questionnaires aimed at the chronically ill but do not address the specific problems experienced by patients with PKU. Recently, a PKU specific HRQoL questionnaire has been developed and has been demonstrated to reliably assess the multifaceted impact of PKU on patients of different age groups [57]. Bosch et al. [58] showed good HRQoL in 306 PKU patients and 253 parents using this PKU specific questionnaire. Negative impacts of PKU on a patient’s life, in particular the emotional impact of PKU and its management (anxiety about blood Phe levels, guilt related to poor adherence to dietary restrictions or Phe-free amino acid supplement intake) was found by the PKU specific HRQoL across all age groups [58].

There are no clinical studies available about the utility of measuring psychosocial functioning in PKU but its usefulness has been studied in other diseases. Measurement (HRQoL instruments) and discussion of psychosocial functioning during clinic visits significantly increased dialogue about psychosocial and emotional function in cohorts of adults, and in children with cancer [167–169], without increasing the duration of the consultation [167, 169]. In other conditions, such as adults with cancer and children and adolescents with diabetes, improved psychosocial outcomes were demonstrated [167, 169, 170], but de Wit et al. showed in diabetes that improvements dissipated after 1 year when measurement and discussion about psychosocial function and wellbeing did not occur [171]. One study evaluating the effect on metabolic control in children with diabetes could not demonstrate improvement [170]. Considering that measurement of psychosocial functioning is usefull in other diseases, this could also be applied to PKU.

In PKU, for evaluation of HRQoL we advise using the PKU QoL questionnaires in addition to generic questionnaires.
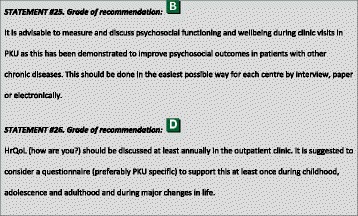


### Mental health problems in early treated PKU

Determining the impact of PKU on mental health is difficult. One of the reasons for this is that different terminology is used e.g. behavioural difficulties, mental health, adaptive issues, and psychiatric symptoms. Adaptive behaviour is more commonly used and this is defined as a collection of conceptual, social and practical skills necessary to function appropriately in daily life. In addition, studies have used different questionnaires to assess mental health. Studies using the Child Behaviour Checklist (CBCL) in ETPKU patients of various ages, report differences in mainly internalizing problems such as social problems and withdrawal, anxiety/depression, poor attention and low self-esteem compared to the normal population [172–176]. However, when these PKU patients were compared to another chronic disorder of childhood, Diabetes Mellitus, no significant differences were found [173, 175]. While Weglage et al. [175] found no correlation between Phe values and CBCL scores, results of Jahja et al. [177] demonstrated that concurrent Phe was correlated to both internalizing and externalizing behavioural problems in children [175, 177]. Jusiene et al. [174] demonstrated that parental emotional coping accounts for 38% of the variance of internalizing problems. The reported internalizing symptoms in ETPKU are mainly attributed to having a chronic illness.

Smith et al. [178] demonstrated an increased prevalence of deviant behaviour strongly related to Phe values in a cohort of 544 PKU patients aged 8 years and 1088 controls. Burgard et al. [179] found more moderate psychiatric disturbances in 60 PKU adolescents compared to 191 age matched controls, although these seemed to be more associated with the chronic condition than with the Phe level. Other studies using personality inventories and depression inventories found no significant differences in mental health between PKU patients and controls or norm scores [112, 180, 181].

Arnold et al. [182] reported a high incidence of attention problems from a chart study, with a strong relationship to Phe levels in the previous year. Twenty-six per cent of the PKU patients used attention deficit hyperactivity disorder (ADHD) medication compared to 6.5% of diabetes type 1 patients (and 5% of the normal population), mostly without formal ADHD evaluation by a psychologist [182]. Lowering Phe by starting BH4 treatment (without dietary adjustments) seemed to decrease ADHD symptoms in 38 patients aged >8 years [183].

Baieli et al. [184] found no patients with autism spectrum disorder in 62 ETPKU patients compared with 2 patients with autism spectrum disorder in 35 late treated PKU patients [184].

In adults with early and continuously treated PKU, there is a lack of evidence about mental health issues. Results from Jahja et al. [177] showed that adult PKU patients presented with more internalizing behavioural problems compared to controls. The Phe levels during childhood were associated with the internalizing behavioural problems [177].

To summarize, in ETPKU children there is an association of internalizing symptoms such as anxiety and depression with elevated concurrent and lifetime Phe levels. The impact of early treated PKU on mental health is most likely to be multifactorial, associated with chronic illness, persistently elevated Phe values, parental coping strategies and executive function deficits. PKU does not seem to enhance the autistic vulnerability in early treated subjects. Whilst studies have detected some increase in symptoms (e.g. anxiety) there is no strong evidence of increased psychiatric disease.

Because of the reported adaptive (behavioural and social) issues in ETPKU, it is important that behavioural screening is incorporated into the follow up of PKU. This enables patients to be referred to the appropriate services should severe difficulties be observed. Although there are many tests, the test choice is largely a professional preference and/or centre dependent.
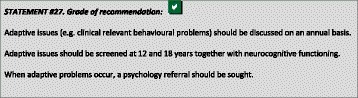


### Oxidative stress

Oxidative stress is described in PKU patients and in PKU animal models as in many (neurodegenerative) disorders. In PKU it could be of importance in our understanding of cerebral PKU pathophysiology. There is clear data suggesting oxidative stress is related to poor metabolic control [101, 185] and micronutrient deficiencies (selenium, zinc, co-enzyme Q10 and perhaps L-Carnitine) [186, 187].

Due to the lack of clinical data linked to anti-oxidant status, no biochemical monitoring is proposed. Good blood Phe control appears to be important in reducing oxidative stress.
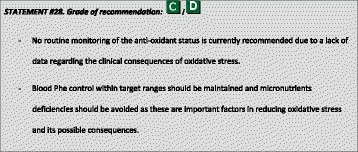


## Dietary treatment

Dietary treatment is the basis of PKU management. It consists of 3 parts: natural protein restriction, Phe-free-L-amino acid supplements, and low protein food. Although we have longstanding experience with dietary treatment, it is only in recent years that there is more scientific evidence to support practice, but there remain gaps in several key areas.

### Natural protein restriction

Phenylalanine is an indispensable, aromatic L-amino acid. It is essential for protein synthesis [188] and so must be provided in an amount that supports growth and tissue repair during childhood, and tissue repair in adulthood while keeping plasma Phe concentrations within recommended ranges [189].

#### Requirements for Phe

In order to promote protein synthesis, it is important to give the maximum amount of natural protein tolerated [190]. In PKU, the individual dietary Phe tolerance is influenced by many factors: severity of PKU, net protein catabolism-synthesis ratio, energy intake, dosage and distribution of Phe-free L-amino acid supplements, and target blood Phe concentrations. The individual Phe tolerance should be pragmatically determined, as minor increases of Phe intake may not necessarily affect blood Phe concentrations [191]. The Phe tolerance is defined as the amount of *Phe per kg of body weight* or *mg/day* that maintains blood Phe concentrations within the target range. This may also be described as natural protein tolerance expressed as *g/day*. In PKU, generally Phe tolerance/requirements per kg of body weight are highest in early infancy ranging from 55 mg/kg/day at 0–3 months of age to 27 mg/kg/day at 12 months [192]. After the age of 1 year, there is a slow and steady decline in tolerance per kg of body weight, and even from the early times of treating PKU with diet it has been recognized that children with classic PKU usually only tolerate between 200 and 500 mg Phe/day. Patients with a milder form of PKU (untreated blood Phe concentrations less than 1000–1200 μmol/l), usually tolerate ≥500 mg/day of dietary Phe. By comparison, in non-PKU, the third US National Health and Nutrition Examination Survey (NHANES III) demonstrated that mean daily dietary Phe intakes for all life stages and gender groups was as high as 3400 mg/day (http://www.cdc.gov/nchs/nhanes.htm). A clear relationship between Phe tolerance at 2 years of age and at 10 years of age was found [40], although it is unknown how this tolerance relates to the tolerance in older patients with PKU aiming to achieve a target blood Phe of 120 to 600 μmol/L. There is clearly a need to evaluate the Phe tolerance of all patients periodically, but particularly at the times of rapid growth, changes in body composition or use of different treatment modalities (e.g. BH4). For patients responsive to BH4, it is likely that natural protein tolerance may double [193] or quadruple [193, 194]. However, there still may be over restriction of dietary Phe intake with low Phe diets (with and without BH4 treatment). Both MacLeod et al. and van Rijn et al. demonstrated in 8 (not clearly well-controlled patients) and 6 (well-controlled) patients that Phe intake could increase substantially without changing the blood Phe concentrations significantly [190, 195]. It has also been shown that Phe intake can be increased with a higher dose of Phe-free L-amino acid supplements [196, 197]. Finally, regular assessement of actual Phe intake, compared with the prescribed amount is important in helping to define actual Phe tolerance as some patients eat more Phe than their prescribed amount without affecting blood Phe control [103, 198].

#### Phe deficiency

Reports of symptomatic Phe deficiency still appear in the literature [80, 81]. Symptoms include: anorexia, listlessness, alopecia, perineal rash, poor and variable growth in preschool children and even death, while biochemical abnormalities include generalized aminoaciduria. Unnecessary dietary restrictions should therefore be avoided.
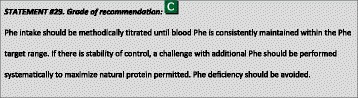


### Protein requirements

In most patients, it is likely that precursor free L-amino acids will supply 52 to 80% of the total protein intake [199–201]. However, the optimal amount of L-amino acids has caused extensive debate and is undetermined. The recent Cochrane review concludes there is insufficient data to reach any conclusions regarding the dosage of Phe-free L-amino acid supplements in the treatment of PKU [201]. Considerations for any recommendations for the dosage of Phe-free L-amino acid supplements intake should include:

1. the protein recommendations for the healthy individual;
2. studies on growth in PKU;
3. inefficiency associated with the utilization of L-amino acids;
4. any functional effects of L-amino acids;
5. any side effects associated with dosage of L-amino acids;
6. natural protein tolerance (i.e. natural protein + Phe-free L-amino acid supplements = total protein intake). The latter will vary according to the severity of PKU, age, clinical condition (e.g. presence of malnutrition/malabsorption), growth, and use of additional treatment options such as BH4.

In addition, the current estimates of protein requirements are defined as the lowest level of dietary protein intake that will balance the losses of nitrogen from the body, and thus maintain the body protein mass in persons at energy balance with modest levels of physical activity [202]. Such a definition does not necessarily identify the optimal intake for health, which is less quantifiable [203], or any specific requirements in clinical situations. To make it even more complex, only the need for L-amino acids as a whole is discussed, but in fact each amino acid (especially indispensable) deserves individual attention, as many of them are large neutral amino acids (LNAA) that may play a specific role in PKU pathophysiology [2, 204].

#### Protein requirements for growth/physiological needs

A number of observational studies (infants to adults) have investigated the Phe-free L-amino acid supplements dosage necessary for optimal growth in PKU [192, 196, 197, 205–220]. They have demonstrated that growth in PKU is mainly satisfactory if the total protein intake (largely given as Phe-free L-amino acid supplements) meets or is above the general population recommendations. In the published studies, national recommendations were commonly based on the FAO/WHO/UNU 1985 safe levels of protein intake [221]. However, recently, the FAO/WHO/UNU 2007 has reduced the safe levels of protein intake (in infants under 1 year by approximately 25 to 27%, children 1–5 years by 17 to 21% and children 6–10 years by 8 to 13%) (Table 3) [202]. No studies have examined growth in PKU on this level of total protein intake so these requirements should not be used until there is published data to support such a low protein intake in PKU. Many centres in Europe and beyond prescribe L-amino acids/total protein between 2 and 3 g/kg/day in infants aged 0–1 y; children aged 1–10 y; 1.5–2 g/kg/day and >10y: 1 g/kg/day (Table 4) [6, 207]. This data was confirmed by a survey of 63 PKU centres from 18 countries, demonstrating that prescription patterns of total protein intake was influenced by country and location in Europe (e.g. South, North, West, East Europe) [222]. In general no more than 20% of energy should be supplied as protein [223].

**Table 3:** A comparison of the protein intakes recommended by FAO/WHO/UNU 1985 and FAO/WHO/UNU 2007 report

Age	FAO/WHO/UNU 2007 Report	FAO/WHO/UNU 1985 Report	% change between 2007 and 1985 safe levels of protein intake
Years	Safe level (+1.96SD)	Safe level (+1.96SD)	
	g/kg/day	g/kg/day	
0.5	1.31	1.75	−25%
1	1.14	1.57	−27%
1.5	1.03	1.26	−18%
2	0.97	1.17	−17%
3	0.90	1.13	−20%
4	0.86	1.09	−21%
5	0.85	1.06	−20%
6	0.89	1.02	−13%
7	0.91	1.01	−10%
8	0.92	1.01	−9%
9	0.92	1.01	−9%
10	0.91	0.99	−8%
Girls			
11	0.90	1	−10%
12	0.89	0.98	−9%
13	0.88	0.98	−10%
14	0.87	0.94	−7%
15	0.85	0.87	−2%
Boys			
11	0.91	0.99	−8%
12	0.90	0.98	−8%
13	0.90	1	−10%
14	0.89	0.97	−8%
15	0.88	0.96	−8%

**Table 4:** International reported protein recommendations for protein equivalent intake (g/kg/day) in PKU

Age y	Brussels Belgium [6]^,a^	Munich Germany [6]^,a^	Copenhagen Denmark [6]^,a^	Madrid Spain [6]^,a^	Milan Italy [6]^,a^	Oslo Norway [6]^,a^	Groningen Netherlands [6]^,a^	Warsaw Poland [6]^,a^	Ankara Turkey [6]^,a^	Birmingham United Kingdom [6]^,b^	Portugal [485]^,c^	United Kingdom (Medical Research Counsil PKU) [486]	France [487]	USA [223]
0.1y	≤2	2–2.3	2–3	3	Depends on Phe tolerance	2.5	2–2.5	2.4	2	3	2–3	3	EAR age	2.5–3.5
1-3y	1.2	1.7	2	2.5		2–2.5	1.8–2	1.6	1.5	3	2–2.5	3	EAR age	≥30 g/d
4-10y	1.2	1.4–1.6	2	2		1.5–2	1.5	1.6	1.5	2	1–2	2	EAR age	≥40 g/d
>10y	1	0.8–1.1	10-14y: 1.5 >14y: 1	1.5		1–1.5	1–1.2	1.2	1.2	10-14y: 1.5 >14y: 1	1–1.5	unreported	EAR age	≥50 g/d to ≥65 g/d
Adult	No data collected	No data collected	No data collected	No data collected	No data collected	No data collected	No data collected	No data collected	No data collected	1	1	unreported	EAR age	≥70 g/d

*EAR* Estimated average requirement

^a^Amount of protein equivalent from protein substitute (g/kg/day); ^b^ Total protein including protein from Phe exchanges (g/kg/day); ^c^ Protein substitute recommendation based on Phe-free L-amino acid supplements rather than protein equivalent

#### Digestibility and bioavailability of L-amino acids

There is insufficient data about the digestibility and bioavailability of L-amino acids [203]. L-amino acid requirements in PKU have not been determined under various conditions such as inadequate energy intake (absorbed L-amino acids may be utilized via catabolism to provide adenosine triphosphate usually referred to as ATP, rather than for body protein synthesis) or on a very low natural protein intake. L-amino acids do not require digestion and are directly available for absorption by the small intestine [224]. This leads to rapid absorption [225, 226]. Not only do plasma amino acids rise more quickly and to higher concentrations but also fall more quickly than whole protein sources like casein [226]. In addition nitrogen retention [227] following ingestion of L-amino acids is less efficacious than with casein rich protein suggesting a less efficient transfer of L-amino acids into tissue and plasma proteins [225, 228]. There is also a suggestion of increased oxidation when Phe-free L-amino acid supplements are taken in large single doses [229], but this may be reduced by small frequent doses of Phe-free L-amino acid supplements, particularly if given bound with intact protein [230].

Overall, it is well established that Phe-free L-amino acids supplements are associated with a lower biological efficiency compared with natural protein sources and some compensatory factor should be considered for this in the protein requirement recommendations. Some have suggested an additional 20% of L-amino acids should be provided to compensate for their inefficiency (Dutch guidelines, unpublished). This was based on several recommended safe levels of protein including FAO/WHO/UNU [221] and [202]. USA recommended dietary allowances (RDA) also proposes an adjustment of approximately 20% to compensate for losses due to digestibility and protein quality for mainly vegetarian diets, but their baseline protein requirements are higher than the 2007 safe levels of protein intake [202].We suggest providing an additional 20% of L-amino acids to compensate for the ‘digestible indispensable amino acid score’ and also a further 20% of L-amino acids to optimize their impact on blood Phe control. This leads to a total of 40% additional L-amino acids, although the optimal dose for this function is undetermined.

For example, if a man with PKU with a body weight of 100 kg (ideal body weight 70 kg) is allocated 6 g/day natural protein, the intake of L-amino acids is calculated as follows: 70 (ideal body weight) × 0.8 (safe level of protein intake) = 56 g/day total protein requirements.

To calculate the L-amino acid requirement: total protein intake (56 g/day) - natural protein intake (6 g/day) = 50 g/day. This is corrected with an additional 40% of L- amino acids from the protein substitute = 50 g/day × 1.4 = 70 g/day.

Ideally, the protein requirements should be based on ideal body weight for height and age. This is particularly important for overweight and obese patients as their total protein intake may be particularly high if based on actual weight only [231].

#### Functional effect of Phe-free L-amino acid supplements

Protein substitutes should supply an adequate source of Phe-free indispensable L-amino acids. It is well established that they decrease blood Phe concentrations [115, 232–234]. It is also noted that blood Phe concentrations increase when patients do not take their Phe-free L-amino acid supplements as prescribed [232, 235]. Higher doses of Phe-free L-amino acid supplements are associated with improved Phe control [233] and a higher Phe tolerance [196, 197]. Some of this has been attributed to improved anabolism associated with a higher dose of L-amino acids but it may also be related to the ability of specific individual LNAA (histidine, isoleucine, methionine, leucine, threonine, tryptophan, tyrosine and valine) within the supplements to alter Phe transport at gut epithelial level [236]. Within regular Phe-free L-amino supplements 35–50% of total L-amino acids are supplied by LNAA’s. Two randomized controlled trials on LNAA demonstrate that they significantly reduce blood Phe concentrations [235, 237, 238]. Large neutral and the cationic (lysine and arginine) L-amino acids cross the intestinal mucosa by a carrier protein similar to that of the blood brain barrier. In in vitro studies investigating intestinal epithelial transport, lysine, histidine, leucine and tyrosine significantly reduce Phe transport [236]. This suggests that the competition with the transport of Phe can be achieved by high concentrations of cationic L-amino acids and LNAA in the gut [236]. There is also evidence to demonstrate that LNAA block the transport of Phe across the blood brain barrier [239–242]. It is therefore established in PKU that L-amino acids within Phe-free L-amino acid supplements do more than provide replacement protein for growth and to maintain body protein mass. They have a role in blood Phe control, they inhibit the transport of Phe into the brain, and possibly via the gut too, and evidence suggests higher doses improve Phe tolerance. In athletes, although exercise is considered to have a major impact on protein metabolism and additional protein may be necessary to support global energy demands, we are unable to give a recommendation on any additional protein requirement for high level sports as this has not been studied in PKU.

#### Adverse effects of Phe-free L-amino acid supplements

Any side effects of Phe-free L-amino acid supplements should be considered. Phe-free L-amino acid supplements are hyperosmolar and so may cause gastrointestinal upset [231]. The osmolality of protein substitutes designed for children range from 600 to 2700 mosmol/kg H_2_0 depending on their dilution with water compared to 300–975 mosmol/kg H_2_0 for paediatric enteral feeding products based on milk protein (data from manufacturers data sheets). Abdominal pain, diarrhoea and constipation have been reported in a small series of young children [243]. Also, life-long and higher intake of Phe-free L-amino acid supplements is linked to proteinuria and decreased glomerular filtration rate in adults [199], although this has not been studied in a controlled way. Future research is needed to determine if this should be part of routine care.

Dental health may be affected by Phe-free L-amino supplements but few studies have examined dental health in PKU. One study reported no significant difference in dental caries between a group of children with PKU and controls with 75% of all children being caries free. However, they did identify more signs of tooth wear in PKU children, associated with the titratable acidity of flavoured Phe-free L-amino acid supplements (92.86–126.8 mEq/l), which is significantly higher than unflavoured supplements (4.18–14.0 mEq/l) and coca cola (38.56 mEq/l) [244].
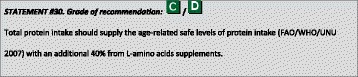


### Supplementation of L-amino acids

In patients with PKU who requires severe restriction of natural protein intake, the provision of a suitable Phe-free protein replacement/substitute is essential to prevent protein deficiency and optimize metabolic control. Protein substitutes are mainly sourced from Phe-free L-amino acids and less commonly from low Phe glycomacropeptide protein. The Phe-free L-amino acid supplements should be evenly administered throughout the day [103, 233] to minimize losses of L-amino acids due to oxidation, and to help minimize fluctuations in blood Phe concentrations over a 24-h period. Therefore, we advise to divide the Phe-free L-amino acids into at least 3 equal portions throughout the day. The dosage and administration of Phe-free-L-amino acid supplementation should be managed by the metabolic dietitian and/or physician.

#### Supplementation of Phe-free L-amino acids with added nutrients

Many Phe-free L-amino acid supplements contain the addition of variable amounts of carbohydrate [245], vitamins, minerals, and long chain polyunsaturated fatty acids (LC-PUFA), with the aim of meeting the nutritional requirements for a product specific age targeted population. The aim is to ensure that the dietary needs for all vitamins, minerals and LC-PUFA are met when average dosages are prescribed for a specific age targeted population. However, higher or lower doses of Phe-free L-amino acid supplements may affect vitamin and mineral intake accordingly. Two longer term observational studies have reported the impact on micronutrient status of Phe-free L-amino acid supplements with added micronutrients [118, 246]. The review by Robert et al. [120] on related studies addressing micronutrient status in PKU suggested that there have been fewer deficiencies since the mid-1990s when the practice of adding vitamins, minerals and trace minerals to Phe-free L-amino acid supplements was increased.

#### Balance of L-amino acids in supplements

In the normal population, there are guidelines for L-amino acid scoring pattern for infants, children and adults [202, 203]. In infants it is considered that breast milk amino acid content is the best estimate of amino acid requirements, but data on requirements from 1 year to adults is unreliable [203] and therefore any requirements for children are estimated using a factorial approach based on L-amino acid requirements for maintenance and growth. The ideal L-amino acid profile of Phe-free L-amino acid supplements is not determined.

#### Presentation of Phe-free L-amino acid supplements

For children over 12 months of age, Phe-free L-amino acid supplements are mainly presented as flavored/unflavored powders (cans/pre-measured sachets) and ready to drink liquids (pouches, bottles and tetrapaks). Powders are designed to be used either as a gel/paste, drink or mixed with food. Additionally, low volume semi-solid weaning products, capsules and tablets are available. Adherence with Phe-free L-amino acid supplements is reported as a major issue, mainly associated with their bitter taste [189, 247, 248]. Failure to take the prescribed amount is linked with poor metabolic control [232, 235]. However, recent evidence suggests that improved taste, volume, presentation and availability (by home delivery) of existing preparations has improved long-term adherence, particularly in teenagers taking liquid Phe-free L-amino acid supplements [246, 249, 250]. Therefore, it is important that patients have a choice of suitable age appropriate Phe-free L-amino acid supplements.

#### Transitioning of Phe-free L-amino acid supplements

Many of the Phe-free L-amino acid supplements are designed for different age groups e.g. Phe-free L-amino acid infant formula, weaning and toddler products, or supplements aimed at school children, teenagers and adults. There are no published studies indicating the best way to support patients transitioning Phe-free L-amino acid supplements from one age group to another. It is established that neophobia is prevalent in children with PKU and many find change particularly difficult [251]. A staged, systematic approach carefully transitioning over products from one to another may be warranted but requires research.

#### Alternative sources of protein substitute

GMP is a low Phe protein source derived from whey protein, used as an alternative to Phe-free L-amino acid supplements in the treatment of PKU. Commercially available GMP protein still requires supplementation with a significant proportion of L-amino acids including tyrosine, tryptophan, histidine and leucine [252] and does contain some Phe. In PKU, human GMP research is limited. Short-term data from a small controlled study in older patients suggest that fasting Phe concentrations and blood urea nitrogen were significantly lower with GMP compared with Phe-free L-amino acid supplements [253]. There is also the suggestion that GMP lowers post-prandial concentrations of the appetite stimulating hormone ghrelin, and may help promote satiety [254]. So far, no long-term outcome data has been reported, and no information about nutritional status or GMP’s safety in children less than 11 years of age has been published. It should not be considered as an alternative source of protein equivalent in children or pregnancy until more data is available.
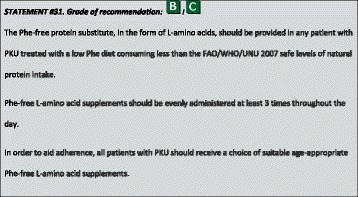


### Nutritional requirements

#### Calorie requirement and energy expenditure

It is assumed that the energy requirement of patients with PKU is similar to healthy individuals, although there is very little supporting published data. Allen et al. reported that resting energy expenditure in children with PKU did not differ from that of normal children and data describing the energy intakes of patients would suggest that they are close to estimated average energy requirements [255]. Rohde et al. [191] found that patients consumed 95% of the RDA as agreed by the German speaking counties (DACH-RDA). MacDonald et al. [256] reported a mean energy intake of 105% of the estimated average requirement, and Rocha et al. [257] reported that patients with classical PKU consumed an additional 100 kcal/daily than patients with mild PKU and 200 kcal/daily more than patients with HPA. There is a growing number of reports of increased incidence of overweight and obesity in PKU [258], but in Portugal and the UK it has been shown to be similar to control groups [257] or general populations [77]. Although there is some association with poor adherence particularly in females with higher blood Phe concentrations [77, 258, 259], currently there is no direct correlation with the consumption of a high carbohydrate foods and overweight and obesity in patients with PKU.

Notwithstanding the importance of avoiding excess energy intake, it is equally important that age related average energy requirements are met for optimal dietary protein utilization, preventing catabolism (resulting in increases of blood Phe) [260–262]. Catabolism is defined as degradative metabolism that breaks down complex molecules as protein or lipids, releasing energy. Protein synthesis and catabolism are energy dependent and thus are sensitive to dietary energy deprivation. Insulin is secreted in response to carbohydrate (and protein) intake, promoting cellular uptake and use of L-amino acids. Energy and/or glucose depletion will result in L-amino acid (especially branched chain) breakdown (gluconeogenesis) to meet minimal glucose requirement, which can ultimately lead to a loss of metabolic control. When energy intake is decreased, protein required to maintain the same nitrogen retention is increased in proportion to the energy decrement [263–265].

Although it is aimed to give a percentage distribution of energy, carbohydrate, and fat similar to recommendations for a healthy population, in a low Phe diet, only 20 to 25% of energy is provided from fat [266, 267] (a typical omnivore diet will provide at least 35% energy from fat). This is due to a low intake of fat/protein containing foods, and increasing carbohydrate to provide almost 60% of energy requirements [256, 257], with 15% from protein equivalent sources. Therefore, a strict low Phe diet is also low in α-linolenic acid, arachidonic acid and without dietary sources of eicosapentaenoic acid (EPA) and DHA [268]. Evidence suggests that children with PKU have reduced concentrations of DHA in plasma and membrane phospholipids when compared to controls [266, 269–271]. Controlled trials with DHA ± arachidonic acid supplements [272, 273] have led to improvement in LC-PUFA status. It is important that consideration is given to supplementation with EPA and DHA if these are not already added to the precursor-free L-amino acid supplement. The optimal dosage of DHA/EPA in children is not established but dosages between 180 and 500 mg daily are provided by Phe-free L-amino acid supplements for children aged between 2 and 16 y.

#### Micronutrient requirement

Micronutrient intake should at least meet theoretical age-related reference nutritional intakes for the normal population. The main micronutrient sources are chemical and usually added to Phe-free L-amino acid supplements. The bioavailability of all micronutrients added to Phe-free L-amino acid supplements is not well studied, and there is little longitudinal data on micronutrient status in patients with PKU following both strict and relaxed dietary regimens.
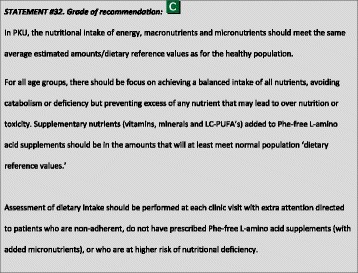


### Low protein foods, fruits and vegetables

A number of low protein foods have been developed for PKU and are important for satisfying appetite and providing variety in a low Phe diet [274]. The availability of low protein foods is a key element in the successful application of a low Phe diet. Many regular basic foods such as bread, flour, and pasta based on wheat flour are not permitted in a low Phe diet because they contain too much natural protein. Instead they are replaced by low protein equivalent foods made from food starches (wheat, potato and maize starch). These special low protein foods should contain Phe ≤50 mg/100 g (equivalent to protein: 1 g/100 g) of dry product. They are an important source of energy, increase dietary variety and aid dietary adherence. However, they should contain no more energy, fat, carbohydrate or sugar than their equivalent natural protein containing foods. Although the energy contribution from these foods has not been formally reported, they may provide 35 to almost 50% of energy intake in severe PKU. All patients should have access to a choice of affordable basic low protein foods (e.g. bread, pasta, cereal, flour, egg and milk-replacements).

In PKU, most fruit and vegetables only yield 30–40 mg Phe per 1 g of protein [275] compared with foods such as milk and cereals that contain 50 mg Phe per 1 g of protein. There is evidence that fruits and vegetables (potatoes not tested) with a Phe content <75 mg/100 g of food do not elevate plasma Phe concentrations [276]. In addition, vegetables containing Phe between 76 and 100 mg/100 g of food do not increase plasma Phe concentrations when eaten in small portions. Also use of similar ‘free’ fruits and vegetables by other countries has not been shown to adversely affect blood Phe control in short-term and longer-term studies [191, 277, 278]. Table 5 provides an overview of these papers.

**Table 5:** Evidence supporting free use of fruit and vegetables containing Phe ≤75 mg/100 g

Reference	N	Fruit/veg criteria	Study design	Change in Phe intake	Blood Phe control	Grade of evidence
MacDonald et al. 2003 [276]	15	Free use of fruit and veg ≤75 mg/100 g Phe Not potatoes	15 week systematic challenge	Mean Phe # Approx 50 mg/day	No impact on Phe control	2−/C
Rohde et al. 2012 [191]	14	Free use of fruit and veg ≤75 mg/100 g Phe	2 week randomised cross-over -trial	Mean Phe # Approx 50–60 mg/day	No impact on Phe control	1−/B
Rohde et al. 2014 [277]	19	Free use of fruit and veg ≤75 mg/100 g Phe	1 year follow up study	Mean Phe # Approx 60–70 mg/day	No impact on Phe control	2−/C
Zimmermann et al. 2012 [278]	50	Free use of fruit and veg ≤100 mg/100 g Phe Not potatoes	Up to 3 year follow up study	Unreported	No impact on Phe control	2−/C

*N* number of patients

Permitting fruits and vegetables without limitations with a Phe content <75 mg/100 g of food will allow greater dietary variety and freedom and will aid dietary adherence. One exception is potatoes. They contain a variable Phe content and their effect on Phe control of their ‘free’ inclusion in the diet requires further testing. 
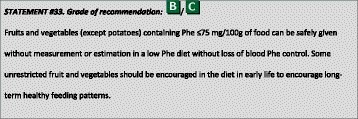


### Breast-feeding

Breast-feeding offers several nutritional, psychological and practical advantages. Generally it is low in Phe (46 mg/100 mL), contains long chain polyunsaturated fatty acids and many non-nutritional bioactive compounds, is convenient, reduces the number of infant bottles, and provides the mother some control over the feeding process [279]. Many studies have reported satisfactory blood Phe control and growth with its use [280–285] and it is advocated by many PKU centres [6].

There have been different approaches to breast-feeding technique. Many have reported demand breast-feeding on the principle of giving a measured volume of a Phe-free infant formula before breast feeds, so reducing stimulation and production of breast milk, thus reducing breast milk and Phe intake. Blood Phe concentrations are used to determine the volume of Phe-free infant formula [279, 284]. Van Rijn et al. [285] alternated feeds between breast-feeding and Phe-free L-amino acid infant formula bottle-feeding and was able to achieve acceptable blood Phe control.
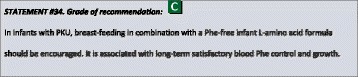


### Aspartame

Aspartame (E951) (1-aspartyl-1-Phe methylester) is an intense sweetener derived from a dipeptide composed of Phe (50%), aspartic acid (40%) and methanol (10%) [286, 287]. It is a source of Phe. It is added to soft drinks, chewing gums, sweets, desserts, jelly and table top sweeteners. The approximate amounts of aspartame in foods are: a 360 ml can of diet coke is 130 mg, 1 portion aspartame flavoured jelly is 40 mg, 1 teaspoon of artificial sweetener 15–20 mg and 1 piece of sugar-free chewing gum 5 mg. In the 1980’s/1990’s, many small intervention (controlled and uncontrolled) studies examined the impact of aspartame in patients with PKU, with most studies demonstrating a small, but consistent, increase in blood Phe concentrations with aspartame [287–295]. Fortunately, there are many other artificial sweeteners available that do not contain Phe. These artificial sweeteners include sucralose, saccharin, and acsesulfame potassium, so aspartame is easier to avoid in a low Phe diet.

The sweetener, neotame, also contains Phe, but the availability of Phe is largely reduced due to inability to break down the peptide bond between aspartic acid and Phe [296].
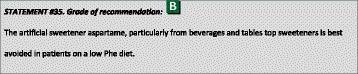


### Tyrosine supplementation

In PKU, Tyr is an indispensable L-amino acid because it is not supplied endogenously via Phe hydroxylation or only to a limited degree. L-tyrosine is important for the biosynthesis of the brain neurotransmitters (epinephrine, norepinephrine and dopamine), thyroxin and melanin skin pigments. Diurnal variations in blood Tyr concentrations are wide with Phe-free L-amino acid supplements that are supplemented with tyrosine. Fasting overnight blood Tyr concentrations are commonly low but then peak immediately following the intake of Phe-free L-amino acid supplements [114], even when given in equal frequent daytime doses. In >80% of 12 PKU subjects, transiently higher than reference range Tyr concentrations occurred although they were not associated with adverse consequences [114, 297]. Therefore, in PKU, Tyr supplementation produces marked but unsustainable increases in plasma Tyr concentrations.

Tyr is added to all Phe-free L-amino acid supplements providing 9 to 11% of their L-amino acids. Therefore, most Phe-free L-amino acid supplements provide approximately 100 mg/g protein equivalent of Tyr which is almost double the concentration found in breast milk, and far exceeds the amount in a normal diet (in natural protein, in general 4% of L-amino acids is from Tyr). A patient consuming 30 g/day protein equivalent from Phe-free L-amino acid supplements will take 3 g/day Tyr, and thereby exceeds the usual recommendations for the healthy population [202, 298]. The USA PKU guidelines suggest the following intake of Tyr: children under 1 y, 1100–3000 mg/day; 1- < 4 y, 2800–3500 mg/day; and 4 y to adult 4000 to 6000 mg/day [102], but it is unclear how this was calculated. The optimum amount of Tyr provided in a low Phe diet is unknown, but additional supplementation in excess of amounts added to Phe-free L-amino acid supplements is not associated with benefit.

To improve neuropsychological functioning, some clinics gave additional Tyr to the amounts added to Phe-free L-amino acid supplements [299]. Meta-analysis of 3 randomized, cross-over, trials [300–302] studying 56 subjects given 2500 mg/day [300] or 100 mg/kg/day [301, 302] of Tyr found that although there was improvement in blood Tyr concentrations, it was not associated with improvements in neurological outcome [303].

Finally, adding additional Tyr powder in a low Phe diet is problematic. Although it has a bland taste, it has a poor solubility leading to uncertainty about the actual amount received when added to liquids; the additional dose required is usually small and so it is difficult to measure with accuracy and administer evenly throughout the day.
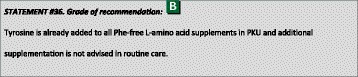


### Large neutral amino acids

Non Phe LNAA include tryptophan, Tyr, histidine, methionine, threonine, leucine, isoleucine and valine. They are considered to have several potential functions in PKU: 1) lower blood Phe by competing with Phe uptake at the blood gut barrier [236–238] although this is not reported by all studies; 2) reducing brain Phe concentrations by providing competition with Phe to cross the blood-brain barrier [240, 242, 304, 305]; 3) increasing cerebral neurotransmitter concentrations (serotonin, norepinephrine, and epinephrine) [242]; and 4) increasing some cerebral large neutral amino acid concentrations such as Tyr, tryptophan, valine, leucine and isoleucine and BCAA [242]. However, in a randomised controlled trial of 16 patients, Schindeler et al. [235] examined the effect of LNAA in combination with diet and Phe-free L-amino acid supplements. They concluded that additional supplementation of LNAA was of limited value, but it may be of benefit in those unable to adhere to their Phe-free L-amino acid supplements [235].

Although some centres routinely administer Phe-free LNAA supplements to older patients who are unable to adhere to dietary treatment, these supplements remained untested in children under the age of 11 years. Their use is also not reported in pregnancy. Overall in PKU, there has been little evidence to support their routine use and further research is required to ascertain the ideal dosages and amount of each specific L-amino acid within the LNAA supplement.
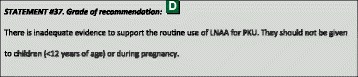


### Illness

It is well established that Phe levels increase during illness. Although patients are not at acute risk, Phe concentrations are likely to remain high until symptoms have abated. In addition, as shown with other IMD, it is known that children experience more illness episodes in the early years when, in PKU, they are particularly vulnerable to the effects of sustained high (and maybe also to fluctuating) Phe concentrations. In general, medical management of illness should be the same as for other children. However, some precautions are of importance as discussed in the next paragraph.

Infection, as occurs in all infants and children, affects their need for and utilization of energy and protein. According to Gardiner and Barbul [306], the ability of the small intestine to absorb L-amino acids is impaired during sepsis. Metabolic changes during infection include increased nitrogen loss, increased need for energy, catabolism of muscle protein leading to elevation of plasma Phe concentrations, conversion of L-amino acids to glucose, and decreased synthesis of acute phase proteins by the liver. Mild to moderate infection increases energy requirements by 20 to 30% [307]. Severe infection increases energy needs by approximately 50% above basal level [308] and it is estimated there is a 13% increase in energy expenditure per degree Celsius of fever [309]. Carbohydrate has been shown to improve nitrogen balance more than the isocaloric amount of fat in catabolic patients on parenteral nutrition [310]. In addition, there is evidence that Phe-free L-amino acid supplements suppress blood Phe concentrations [234]. Therefore, we consider it important that Phe-free L-amino acid supplements and high carbohydrate drinks are administered during infection to help decrease muscle protein loss and potentally lessen impact on deteriorating blood Phe control, although this remains unstudied. The importance of lowering natural protein during each illness episode is also unclear, although lowering natural protein during illness episodes may be necessary in patients treated by diet and/or BH4 [311]. Antipyretics should be administered for high temperatures. Treatment with antipyretics/analgesics like paracetamol and ibuprofen should be considered to improve food, fluid and energy intake during illness. Table 6 summarizes dietary advice for illness.

**Table 6:** Dietary advice for illness in PKU

Diet	Dietary advice
Phe-free L-amino acid supplement	Maintenance of Phe-free L-amino acid supplement intake to support protein synthesis. It is better to give smaller, frequent doses throughout the day.
High carbohydrate intake	Encourage frequent high carbohydrate supplements, e.g. glucose polymer solution.
Natural protein intake	In practice, a reduced appetite leads to a lower natural protein intake.
Medications	All treatment specific medication should be continued during illness. Continue BH4 if already prescribed. Medications should be free of aspartame in PKU.
Treat precipitating factors	e.g. anti-pyretics for pyrexia, antibiotics (aspartame-free) for bacterial infections.

For any illness, if suitable aspartame-free medication cannot be sourced, it is better to use aspartame-containing medications (for example antibiotics) rather than leave a child without treatment. Special attention should be given to ensure routine vaccination according to international/national standards. Gastroenteritis leads to a catabolic status. In young infants, oral vaccination against rotavirus is possible and recommended in most countries.
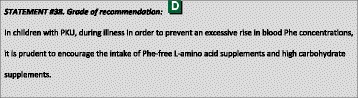


### Parenteral nutrition

There is very limited information about the management and outcome of patients with PKU requiring treatment with parenteral nutrition. Infants, particularly those who are premature, and young children requiring long-term parenteral nutrition, are likely to be at risk from permanent damage if blood Phe concentrations cannot be controlled. Single case studies of premature infants with PKU given standard preparations of amino acid intravenous solutions for limited periods have had very high blood Phe levels although apparently without adverse effects on long-term neurological outcome [312–314]. A specially prepared Phe-free intravenous amino acid solution has been used in a 6 year old with PKU with an intra-abdominal malignancy, which effectively prevented high Phe levels [315]. A commercially available preparation with a lower content of branched chain amino acids designed for use in hepatic failure was used in a premature infant with PKU as this preparation also contained less than usual Phe [312] and in a child with a facial tumour [316]. However elevated Phe levels could not be completly prevented in these cases.

### Support

Living with a life-long severe dietary restriction may adversely affect eating attitudes and behaviours and increase susceptibility to the development of eating disturbances [317]. Coping with and adhering to dietary treatment has been described as a stressor to both the patient and the family. Feeding behaviour problems are more common in young children with PKU [115, 243] and appear associated with the management of feeding behaviour rather than intrinsic to the condition. Food neophobia is also more prevalent in children with PKU, with children being particular about their food choices and untrusting of new foods when compared with control children without PKU [251]. However, early intervention, working alongside psychologists and play therapists, can play an important role in improving feeding behaviors and family mealtime interactions. Strategies that are used for general feeding problems apply to children with PKU e.g. positive caregiver role modelling, gradually increasing familiarity with new foods, consistent mealtime routines with adequate time allocation for eating.

It is commonly reported that children may need constant coercion to take their Phe-free L-amino acid supplements which is exhausting for caregivers and some may resort to strategies such as yelling, grounding or taking away privileges [247]. A study of feeding problems in young children indicated that almost 50% had difficulty with its administration and all children had been given Phe-free L-amino acid supplements since early infancy [243]. Some of these problems may have been related to the developmental age of children or consistency of approach by caregivers. Overall, there is lack of research on the best strategies to support caregivers in the maintenance of administration of Phe-free L-amino acid supplements.

In adolescents and adults with PKU the occurrence of eating disorders has not been systematically explored and rarely reported so may be undetected and untreated. This is an area that requires further study in PKU. Regular health professional support, especially from a psychologist, may provide some measure of protection.
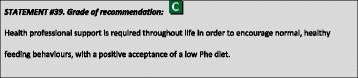


## Treatment in specific patient groups

### Maternal PKU

PKU treatment aims to prevent maternal PKU syndrome [318]. High blood Phe levels during pregnancy have a teratogenic effect on the developing foetus that can result in growth retardation, microcephaly, intellectual disabilities and birth defects, including congenital heart defects (CHD) [318, 319]. When treatment recommendations are adhered to, the chances of a good outcome are comparable to the normal population.

#### Risk of CHD

Lenke and Levy [318] reported CHD in 27 infants of mothers with assigned blood Phe levels (APL) of >1200 μmol/l and in 7 infants when blood Phe levels were between 900 and 1200 μmol/l. These APL were selected as the highest of 2 or 3 plasma Phe levels [318]. In the Maternal PKU Collaborative Study (MPKUCS), Levy et al. [320] described 34 offspring with CHD from pregnancies in women with PKU and in 1 offspring from a mother with MHP. All PKU mothers had APL >900 μmol/l and did not achieve metabolic control before the 8th week of gestation [320]. In a further report from the MPKUCS, Platt et al. [321] described 31 offspring with CHD. When maternal blood Phe levels were 120–360 μmol/l during the first 8 weeks of gestation, no cases of CHD were described; when Phe levels were 360–600 μmol/l, there was one case of CHD; when Phe levels were 600–900 μmol/l, there were 5 cases of CHD; and when Phe levels were >900 μmol/l, there were 26 cases of CHD [321]. Table 7 provides the percentages of offspring with CHD.

**Table 7:** Percentages of offspring with congenital heart disease with Maternal Off-Diet Phe Levels (μmol/L)

	≥1200 μmol/l	900–1200 μmol/l	600–900 μmol/l	180–600 μmol/l	Control group/normal population
Lenke and Levy 1980 [318]	12% of *n* = 225	15% of *n* = 46	6% of *n* = 33	0% of *n* = 44	0.8% in normal population
MPKUCS Koch 2003 [319]	11% of *n* = 257	5%	3%	2% of *n* = 66	1% CHD of *n* = 100 control pregnancies
		*n* = 91 with 600–1200 μmol/l			

In untreated or non-optimally treated pregnancies, increased frequencies of intrauterine growth retardation (IUGR), intellectual disability, microcephaly [318, 319, 322] and other congenital abnormalities have been described in the MPKUCS [323] and case reports (Table 8).

**Table 8:** Reported malformations in the literature

Malformations	
Congenital heart defects - Tetralogy of Fallot - ventricular septal defect - mitral/aortic stenosis - patent ductus arteriosus	
Dysmorphology - microcephaly - coloboma - malformed eyelid, ptosis - hypertelorism - cleft palate - malformed ears - simian creases - fused digits - widely spaced toes	
Other: - anencephaly - oesophageal atresia - renal agenesis, Potter syndrome - hypospadias - hydrocele - anal fistula, anal atresia	

Reported in the MPKUCS [323] and case reports

#### No treatment

For PKU women of childbearing age, no treatment is necessary when untreated blood Phe levels are <360 μmol/l. Levy et al. [324] showed that mean untreated blood Phe levels <400 μmol/l had no effect on birth measurements and no effect on offspring IQ. Platt et al. [321] confirmed no increased rate of birth defects in (un)treated blood Phe levels between 120 and 360 μmol/l. Levy et al. [325] even demonstrated that there was no correlation between the offspring’s IQ and mothers untreated APL <600 μmol/l. Waisbren et al. [326] reported that the offspring of women with untreated MHP had cognitive and behavioural development similar to control subjects.

#### Treatment goals/target Phe levels

Woman with PKU should start a Phe-restricted diet before conception. Many features of the maternal PKU syndrome are preventable by starting a low Phe diet before conception or early in pregnancy [319, 320, 327–329]. Children born to mothers with PKU who attain satisfactory blood Phe control before or very early in pregnancy appear to begin life with normal potential. Maternal delay in attainment of acceptable blood Phe control is associated with decline in offspring developmental outcome/IQ scores [326, 329, 330].

In prospective studies, there is no effect on infant birth measurements and final IQ when mother’s mean blood Phe levels are <360 μmol/l. The MPKUCS study indicated that the major factors associated with good child outcome was normal maternal intelligence and well-treated pregnancies with blood Phe control between 120 and 360 μmol/l [319, 322, 326, 329]. Widaman [51] demonstrated a threshold effect of a mother’s average blood Phe value of 400 μmol/l in relationship to offspring’s IQ. With every further increase of 60 μmol/l Phe, the IQ drops by 4.7 points [51]. In addition it seems to be important that blood Phe concentrations are maintained consistently even within target range [330]. The prevention of CHD requires initiation of the low Phe diet before conception or early in pregnancy (<8th week) [320]. Dietary management that is too strict may be associated with a risk of IUGR in the offspring as described by Teissier et al. [331]. As IUGR is related to an increased risk of diabetes, cardiovascular disease and hypertension later in life, Phe levels below 120 μmol/l should be avoided.

As women with untreated Phe levels between 360 and 600 μmol/l need to return to dietary treatment prior to conception, some may consider that during child bearing years, women should continue a small dose of Phe-free L-amino acid supplements to help retain acceptance of its taste, but this practice remains unproven.
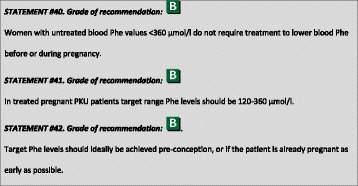


For optimal pre-conception treatment, stable blood Phe control within target range should be maintained before PKU women conceive. The time to reach stable and acceptable blood Phe concentrations varies between women. It is influenced by personal conditions (organizational skills, IQ, work conditions) and family support, which will affect the ability to adhere to strict long-term diet. In general women planning pregnancy are well motivated. The timing of medical consent to stop contraceptive strategies varies between centres and countries. In some European centres, women with blood Phe levels within target range for as little as 2 weeks may be advised to stop contraceptives, other centres may advise several months. We recommend contraceptive strategies should only be discontinued after stable Phe levels within target range have been achieved for a minimum of 2 weeks. There is no evidence that maintaining blood Phe levels within target range for a period of 2–3 months vs 2 weeks pre-conception is associated with better outcome. Shorter pre-conception periods may help maintain patient motivation. However, achieving ‘blood Phe levels within target for at least 2 weeks’ may take several weeks as some women with PKU need time to adapt to the rigorous demands of a low Phe diet and obtain regular and consistent access to special dietary products.

#### Pregnancy planning and medical follow-up

Maternal PKU is considered a high risk pregnancy as it is difficult to prevent high or low blood Phe concentrations, necessitating follow-up by a obstetrician well informed about PKU as well as a metabolic dietician and metabolic physician. Minimal outpatient clinic visits of once during each trimester is recommended, but many health practitioners may advocate more frequent follow-up and the intensity of monitoring will depend on individual needs and metabolic control.

Metabolic control is based on weekly Phe blood spots pre-conception and at least twice weekly during pregnancy, with speedy laboratory turnaround times.

PKU women should undertake the same pre-conception screening as recommended for healthy women and should receive education about healthy lifestyle and behaviour. These recommendations are found in the (inter)national maternity guidelines [332].

As maternal PKU treatment needs considerable effort from the woman and her partner, failure to conceive requires special attention. Therefore, it is reasonable to refer well-controlled patients to a fertility specialist earlier than after 1 year (the WHO criteria of sterility) and it seems appropriate to recommend a time period of 6 months [333].

In maternal PKU there are 2 main risks for fetal development: growth retardation and birth defects including CHD. Therefore, detailed follow-up by ultrasound examination specific for high risk pregnancies (especially for inadequate metabolic control) is highly recommended from the very early beginning of pregnancy with screening for organ development at 18–22 weeks of pregnancy [320, 334].

Post delivery women with PKU should receive routine obstetric care and should be encouraged to return to standard dietary or pharmacological treatment [335]. There is a relationship between postnatal child stimulation in the home environment and their developmental outcomes [326]. There are no studies available about Phe toxicity of the mother post-delivery and the risk of postnatal depression.
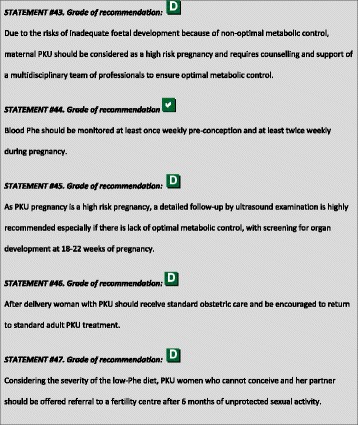


##### Offspring follow up after delivery

Infants suffering from a birth defect or severe health problems must be treated after birth. An echocardiogram should be considered in all infants who are conceived by women with high blood Phe levels and in those with poor maternal blood Phe control during pregnancy [333]. After sub-optimal pregnancy treatment, infants commonly have smaller birth measurements and delayed cognitive development. Children should be followed up, preferably in specialized centres, similarly to other ‘at risk’ infants e.g. preterm infants or small for gestational aged children.

#### Prevention of unplanned pregnancies

Unplanned pregnancies in woman with PKU are a significant health problem [336]. In 2008 in Europe, in the general population 44% of conceptions were unplanned [337]. Prevention of the maternal PKU syndrome requires ongoing education from childhood into adulthood about foetal risks associated with high plasma Phe concentrations and importance of pregnancy planning [338, 339]. This information should be re-inforced by all members of the core PKU team. Patients with PKU also consider that counselling and education on the dangers of unplanned pregnancy is required [11]. For women with untreated Phe levels <600 μmol/l who may have stopped diet in adolescence, a robust transition process between paediatric and adult services is essential to ensure they are not lost to follow-up. Clinic patient registries and on-going contact with the PKU team are important.

PKU women at childbearing age, with all forms of HPA, should receive detailed counselling regarding family planning and the risks of adverse foetal effects as a consequence of elevated Phe levels. If teenage PKU girls and women are suspected of sexual activity, the most effective birth control methods should be advised. In 60 PKU women, factors associated with contraception usage were the extent to which women felt socially supported to use contraception (*r* = 0.64) along with positive attitudes about birth control (*r* = 0.66) and knowledge of family planning (*r* = 0.43) [340].

From the age of 12 years (beginning of puberty), all patients should receive systematic age-related sex education, with professional counselling about the risk of unprotected sexual contacts. They should be informed that unplanned pregnancy can occur even during the first menstrual cycle. At least one PKU team member should be able to provide sex education information. Pre-conception education initiatives that have been developed in for example diabetes could be adapted for PKU care [341, 342].

It is important that all advice is individually tailored, particularly for women with psychological and/or intellectual impairment. Individual cultural and religious background should be considered in patient counselling.
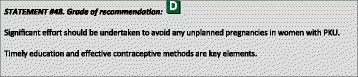


#### Nutritional recommendation in maternal PKU

The nutritional status of women with PKU both during pre-conception and pregnancy is likely to have significant influence on foetal and infant outcomes. Attentive and proactive management is essential. Maternal weight loss, over restriction of Phe intake, lack of folic acid, and vitamin B12 may all affect foetal outcome. Practical education of women giving them the skills and knowledge to manage their dietary treatment is a key component of care.

##### Phe tolerance

Maternal Phe tolerance is influenced by the severity of PKU but will vary even between pregnancies in the same patient according to adherence with Phe-free L- amino acid supplements, adequacy of energy intake and changes in weight, trimester of pregnancy, and existence of foetal PKU. Reported Phe tolerance is given in Table 9. If the foetus has PKU, it has been reported that Phe tolerance barely increases in the second and third trimester of pregnancy [343]; thereby a low Phe tolerance in the third trimester of pregnancy may indicate foetal PKU [343].

**Table 9:** Reported Phe tolerance during pregnancy in maternal PKU

Reference	Number of patients	1st trimester	2nd trimester	3rd trimester
Vockley et al. 2014 [102]	0 (USA guidelines)	265–770 mg/day	400–1650 mg/day	700–2275 mg/day
Acosta et al. 2001 [350]	240	456 ± 233 to 684 ± 413 mg/day	528 ± 269 to 528 ± 269 mg/day	938 ± 542 to 1248 ± 513 mg/day
Thompson et al. 1991 [351]	1	6 mg/kg bodyweight/day		30 mg/kg bodyweight/day
Kohlschutter et al. 2009 [343]	3	400 mg/day		1700 mg/day (non-foetal PKU) maximum 600 mg/day (foetal PKU)
Duran et al. 1999 [232]	5	250–500 mg/day	300–500 mg/day	
Rohr et al. 1987 [369]	3	450–800 mg/day	720–1300 mg/day	1300–1500 mg/day

From the second trimester onwards a period of rapidly increasing Phe requirement begins owing to foetal-maternal anabolism. As Phe is an essential L-amino acid, it is important that dietary Phe intake is increased (by 50 to 100 mg/day) without delay if blood Phe concentrations are ≤120 μmol/L. Slow increases in natural protein intake may prolong the length of time blood Phe concentrations are <120 μmol/l. Teissier et al. reported from 155 pregnancies in 86 PKU women that Phe intakes were lower in a group with IUGR from the fifth to the 8 month of pregnancy. The longer the duration of time blood Phe below 120 μmol/l, the higher the risk of IUGR [331].
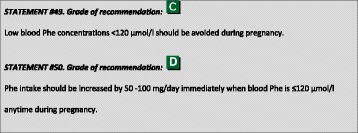


##### Weight gain and energy requirements

Low energy intake, accompanied by weight loss is common, particularly in the first trimester of pregnancy in PKU and this is associated with higher blood Phe concentrations [344]. Inadequate energy intake may be due to dislike of low protein foods, poor adherence with lack of Phe-free L-amino acid supplements, limited availability of low protein foods, inability to prepare low protein meals or poor appetite associated with nausea and vomiting. Poor maternal weight gain (less than 70% of recommended) and foetal microcephaly are correlated [345]. Microcephaly significantly decreases when maternal weight gain is adequate [345]. There is also evidence that inadequate gestational weight gain is associated with decreased foetal growth and birth weight both in the general population [346] and in PKU [347, 348], although further study is required. Data from a French survey reported in 135 pregnancies that the BMI of mothers was lower than the general population but there was no direct correlation with IUGR in children [349]. In a single adult centre in London, women with PKU lost weight in the first trimester and had a weight gain below that recommended for pregnancy [330], probably reflecting a low energy intake.

In the MPKUCS, maternal energy intake was significantly and negatively correlated with plasma Phe concentrations during the last 2 trimesters of pregnancy [350]. In addition, case studies have described difficulty in maintaining blood Phe control due to weight loss and low energy intake in the first trimester [347, 351].

Energy requirements vary considerably for individuals but they should be tailored to pre-pregnancy BMI, rate of weight gain, maternal age, gestational stage of pregnancy, physical activity levels and blood Phe control. Any additional energy costs associated with the maintenance of a normal pregnancy are due to greater maternal and foetal-placental tissue mass, increased energy expenditure attributable to increased basal metabolism and changes in the energy cost of physical activity [352, 353]. Published guidelines for normal maternal energy requirements do vary between ‘expert’ groups and are given in Table 10. Most dietary studies in well-nourished women in non-PKU pregnancy have shown no or only minor increases in energy intake that only partially cover the estimated energy cost of pregnancy [353]. The best way to determine if energy needs are being met is to carefully monitor maternal weight change in pregnancy.

**Table 10:** Additional energy requirements in general population (non-PKU) pregnancy

References	Additional energy requirements in pregnancy (kcal/day)		
	1st trimester	2nd trimester	3rd trimester
UK SACN (2011) [353]	None	None	191 kcal/day
FAO/WHO/UNU (2001) (based on gestational weight gain of 12 kg) [393]	85 kcal/day	360 kcal/day	475 kcal/day
^a^IOM dietary reference intakes (2005) [382] For women 19–50 years	None	340 kcal/day	452 kcal/day
^a^IOM dietary reference intakes (2005) [382] for girls 14–18 years	None	340 kcal/day	452 kcal/day

^a^Energy requirements based on following assumptions: total energy expenditure changes little and weight gain is small during the first trimester so additional energy recommended during second and third trimester only

Birth weights between 3.1 and 3.6 kg (mean, 3.3 kg) are associated with the optimal ratio of maternal and foetal health outcomes [354]. This is in turn associated with a pregnancy weight gain of 10–14 kg (mean 12 kg) [354].

If there is maternal weight loss, additional energy from low protein foods (e.g. pasta, bread) and energy supplements (glucose polymer/fat emulsions) should be considered, and weight monitored weekly until weight gain is satisfactory [355]. However, excess weight gain should also be prevented as maternal obesity is associated with additional complications throughout pregnancy and increased health risks to the mother and her infant [356, 357]. Overall, in maternal PKU weight gain should be similar to the healthy population (Table 11) with emphasis on avoiding weight loss particularly in the first trimester of pregnancy.
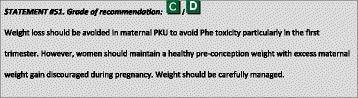

**Table 11:** The Institute of medicine (USA) and National Research Council (USA) Committee guidelines for pregnancy weight gain in the general population [488]

Pre-pregnancy BMI^a^	Total weight gain kg	Rates of Weight Gain^+^ 2nd and 3rd Trimester kg
		Mean (range) in kg/week
Underweight (<18.5 kg/m^2^ )	12.5–18	0.51 (0.44–0.58)
Normal weight (18.5–24.9 kg/m^2^ )	11.5–16 kg	0.42 (0.35–0.50)
Overweight (25.0–29.9 kg/m^2^ )	7–11.5 kg	0.28 (0.23–0.33)
Obese (≥30.0 kg/m^2^)	5–9 kg	0.22 (0.17–0.27)

^a^ *BMI* body mass index, Adolescents should aim for weight gains at upper end of recommendations. ^+^Calculations assume a 0.5–2 kg weight gain in the first trimester

##### Protein requirement

Additional protein requirement during pregnancy is due to newly deposited protein and the maintenance costs associated with increased body weight [202]. The recommended additional protein intake recommended by FAO/WHO/UNU [202] in non-PKU pregnancy is 1 g/day during the first trimester, 10 g/day during the second trimester and 31 g/day during the third trimester, with an efficiency for protein utilization estimated to be 42%. In maternal PKU, total protein requirements are not accurately defined and can only be extrapolated from non-PKU maternal requirements. Reports of protein prescription from Phe-free L-amino acid supplements (with or without natural protein) have varied widely: reference nutrient intake (RNI) + 15% [355], ≥70 g/day total protein [102], and 100 g/day amino acids [358]. There are no reported studies examining the utilisation of Phe-free L-amino acid supplements in specifically maternal PKU.

However, it is established that the delivery of the prescribed amount of Phe-free L-amino acid supplements is important during pregnancy. In the MPKUCS study, lower total protein intakes (<USA RNI) were associated with worse blood Phe control [348]. In another report, mothers who had an inadequate protein intake (less than 50% of recommended amounts) in the first trimester mainly due to nausea and vomiting, together with poor metabolic control, had a higher risk of CHD compared to mothers who had an adequate protein intake and similar blood Phe concentrations [345, 359]. Lower maternal total protein intakes were negatively associated with birth weight and length of the newborns [348]. Lower concentrations of the amino acids: proline, valine, methionine, isoleucine, lysine and arginine in PKU mothers have also been linked with low intake of Phe-free L-amino acid supplements and a higher risk of CHD in their offspring [360]. Inadequate intake of micronutrient supplemented Phe-free L-amino supplement has been associated with low intakes of vitamin B12 and this is also associated with an increased risk of CHD [359].

Duran et al. demonstrated the impact of good adherence with Phe-free L-amino acid supplements on lowering blood Phe concentrations in pregnant women [232].

We have adopted the recommendation that in maternal PKU, a total protein intake of ≥70 g/day is required, but it is also important to consider individual patient weight and the additional protein requirements of each pregnancy trimester. In maternal PKU, more data is required about prescribed and actual total protein intakes compared with infant outcome measures. The protein substitutes based on LNAA [361] and glycomacropeptide have not been reported during pregnancy and are thereby not recommended for maternal PKU until their safe use is established.
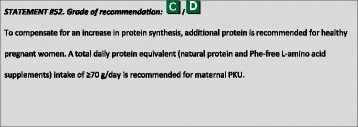


##### Tyrosine supplementation

Many PKU centres advocate additional Tyr supplementation at some stage during pregnancy [333, 347, 351, 362–371], although the specific requirement in maternal PKU is unknown. The amount of Tyr supplementation given is highly variable and usually without justification for the dose chosen (Table 12). Normally, Tyr is a non-essential amino acid synthesized from Phe. In PKU, Tyr cannot be synthesized from Phe, resulting in lower blood Tyr concentrations. It has been proposed that some of the birth defects seen in maternal PKU may be related to low Tyr concentrations [372], and that Tyr supplementation during pregnancy may help improve foetal outcome [370].

**Table 12:** Reported Tyr supplementation during pregnancy in maternal PKU

Author	Tyr supplementation
Coutts 1979 [358]	>10 g/day
Singh et al. 2014 [361], Vockley et al. 2014 [102]	6000 to 7600 mg/day
Rohr et al. 1987 [369]	Up to 6 g/d (did not ↑ blood Tyr >30 μmol/l)
Davidson et al. 1989 [364]	6.4–11.9 g/day
Brenton et al. 1996 [363]	2 g/day
Maillot et al. 2007 [333]	8 g/day (total from Phe-free L-amino acid supplements and Tyr supplements)
Thompson et al. 1991 [351]	160 mg/kg/day- starting point and then titrate according to Tyr levels

In non-maternal PKU, it has been established that Tyr supplementation raises blood Tyr [303]. In maternal PKU, Tyr supplementation raises maternal Tyr concentrations above the recommended minimum concentration (>45 μmol/l) for a period of ≥3 h and is associated with a markedly increased ratio of Tyr to LNAA [370]. However, Tyr supplementation leads to variation in blood Tyr between fasting and fed state, and Tyr concentrations >200 μmol/L have been observed at some time points within a 24-h period [297]. It is also suggested that foetal Tyr concentrations in blood will be some 1.8 to 3.3 times higher than in the maternal blood [297, 373]. A toxic effect of a combination of mildly increased Phe and Tyr was demonstrated experimentally in rats [374].

The FAO/WHO/UNU 2007 suggests the aromatic amino acids (Phe/Tyr combined) requirements for healthy adults are only 30 mg/g protein [202]. Therefore, women taking 75 g/day total protein would require 2250 mg Tyr daily. All Phe-free L-amino acid supplements contain Tyr. The amounts of Tyr in 60 g/day protein equivalent from Phe-free L-amino acid supplements varies between 5 and 7 g/daily and is well in excess of daily requirements. Thereby, this would suggest that any additional Tyr to what is already provided by the L-amino acid supplements is unnecessary. However, in PKU due to aberrant Phe/Tyr metabolism, it may be inappropriate to compare free Tyr requirements to either the FAO/WHO/UNU 2007 aromatic amino acid or protein requirements.

Furthermore, the MPKUCS study found no relationship between infant outcomes and maternal blood Tyr concentrations before and during pregnancy [319]. An optimal range for plasma Tyr concentration in maternal PKU has not been established. It is important that excessive Tyr supplementation should be avoided as the safety of Tyr supplementation during pregnancy has not been extensively studied.
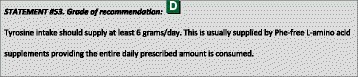


##### Emergency diet during unplanned pregnancies

Even in women with an unplanned pregnancy and high blood Phe concentrations it is essential to reduce Phe intake, although this should not be accompanied with weight loss so avoiding catabolism. Women who become pregnant without appropriate blood Phe control will need significant support to attain Phe levels within the recommended target range in a timely fashion (Dietary education programmes for maternal PKU section). Women should be given emergency supplies of Phe-free L-amino acid supplements and low protein foods until their own supply can be established through the health or insurance systems. Baseline blood Phe concentrations, anthropometry, nutritional biochemistry should be established. The teaching of home blood Phe sampling is necessary.

As a starting point, the initial Phe allocation should be the same as the Phe tolerance when aged between 1 and 5 years of age and will depend on the severity of PKU. In the absence of this information, a guideline for women’s predicted Phe tolerance at the start of pregnancy is suggested by Maillot et al., (Table 13) and Phe intake should then be adjusted according to blood Phe levels [333].

**Table 13:** Predicted amount of Phe tolerated during early pregnancy according to blood Phe concentration on an unrestricted diet (modified from Maillot et al. 2007 [333])

Blood Phe concentration (μmol/L)	Initial amount of daily Phe to give at the start of dietary treatment mg/day
>2000	150
1600–2000	200
1200–1600	300
1000–1200	300

Data show that uncontrolled maternal blood Phe levels (over 600 μmol/l) over 10 weeks of pregnancy result in a high risk of a child developing severe clinical disabilities (Tables 7, 8). Although the risks of CHD are within the first 8 weeks of pregnancy, the risk of impaired foetal development in general and especially to the brain is ongoing throughout pregnancy. Therefore, in such cases, non-directive counselling should be given, with the clinician informing about the high risk (15%) of birth defects (minor or severe). A foetal heart ultrasound is recommended at 18–22 weeks gestation.
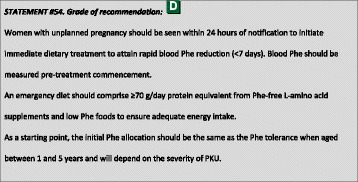


##### Nausea and vomiting/hyperemesis

Nausea and vomiting affects up to 85% of all pregnancies [375]. This may affect the dietary intake and weight gain so should be aggressively treated. In PKU this can lead to elevated blood Phe levels. Symptoms generally begin around week 5 of gestation and typically stop by week 12. Up to 15% of pregnant women experience persistent symptoms until delivery. Hyperemesis gravidarum (affecting between 0.5–2% of women) represents the extreme end of the spectrum associated with dehydration and weight loss greater than 5% of pre-pregnancy weight. Besides nasogastric tubes, gastrostomy tubes have been used in ‘healthy’ pregnant women with hyperemesis gravidarum but are associated with infections and surgical risks [376]. Gastrostomy tube placement is also an option that has been used to deliver Phe-free L-amino acid supplements to women who are unable to restart diet due to severe nausea or palatability issues [377].

The treatment should be tailored to the individual but should include similar but adapted advice to non-PKU pregnancy. Useful additional advice in maternal PKU can be found in Table 14.
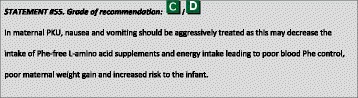

**Table 14:** Advice to help nausea and vomiting in maternal PKU

*Dietary and lifestyle approaches*	Small frequent low protein meals and snacks that are high in carbohydrate (e.g. low protein toast, crackers) and low in fat to avoid an empty stomach, feelings of hunger, and abdominal distension [375]. Cold meals may be better if nausea is associated with food smells. For women who have difficulty in eating solid foods, additional drinks of cold water supplemented with glucose polymer may be tolerated if sipped throughout the day. Women should avoid lying down immediately after meals.
*Phe-free L-amino acid supplements*	Give Phe-free L-amino acid supplements chilled and encourage up to 5 or 6 times during the day in small doses. The high osmolality of Phe-free L-amino acid supplements may aggravate nauseas [347] and so may be better tolerated if given with extra fluid. If the smell of liquid or powdered Phe-free L-amino acid supplements is not tolerated, Phe-free L-amino acid tablets are worth consideration.
	Any doses of Phe-free L-amino acid supplements lost through vomiting should be re-given. In extreme cases of vomiting and Phe-free L-amino acid supplements intolerance, hospital admission and administration of Phe-free L-amino acid supplement via a nasogastric tube could be considered.
*Medication*	Safe antiemetic therapy and acid reducing medications should be considered with persistent vomiting and symptoms of dyspepsia and indigestion.

##### Folic acid supplementation

Little attention has been paid to folic acid intake in maternal PKU. Folic acid is of critical importance both pre- and peri-conceptionally in protecting against neural tube defects in the developing foetus (first 28 days of pregnancy). There is now conclusive evidence from a number of randomized controlled trials that folic acid supplementation can prevent neural tube defects [378–380]. Folic acid requirements increase during pregnancy and many countries give an additional 400 μg/day pre-conceptually and during the first 12 weeks of pregnancy (Table 15). The upper tolerable limit of folic acid in the healthy women recommended in Europe [381] and USA [382] is 1 mg per day. However ‘Diabetes UK’ (www.diabetes.org.uk) recommends 5 mg/day of folic acid in diabetes to prevent neural tube defects due to higher risk.

**Table 15:** National folic acid requirements in general population (non-PKU) pregnancy [489]

Country	Average nutrient level μg/day	Individual nutrient level μg/day
Australia and New Zealand^a^	520	600
Austria, Germany and Switzerland^a^		600
Denmark, Ireland and Sweden^a^		500
European Community		400
Mexico		750 (safe level)
Poland^a^	520	600
The Netherlands^a^		600 (safe level)
USA^a^	520	600
UK^a^		300
FAO/WHO/UNU	520	600 (safe level)

^a^400 μg folic acid supplementation given in addition to folic acid requirements

Whilst Phe-free L-amino acid supplements contain folic acid, the amount is variable and adherence with Phe-free L-amino acid supplements may be poor, resulting in sub-optimal intake. Giving an additional 400 μg/day of folic acid in the early weeks of pregnancy should apply in PKU as in healthy women. Vitamin B12 (including functional marker plasma homocysteine and/or methylmalonic acid) should be monitored to ensure that high intake of folic acid does not mask vitamin B12 deficiency [383].
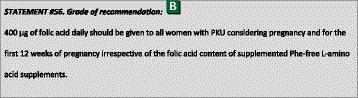


##### EPA and DHA

Sub-optimal concentrations of EPA and DHA have been reported in maternal PKU [384] and traditional dietary treatment is low in α-linolenic acid, arachidonic acid and without sources of EPA and DHA [268]. DHA (n-3) and arachidonic acid (n-6) are essential for foetal growth [385]. In healthy women, an increased supply of n-3 LC-PUFA during pregnancy reduces the risk of preterm birth before 34 weeks of gestation. Pregnant women should achieve an additional supply ≥200 mg DHA/day, over and above the intake recommended for adult general health usually achieving a total intake ≥300 mg DHA/day [386], and this should be given to all women considering pregnancy and during pregnancy in PKU. Many (but not all) Phe-free L-amino acid supplements contain supplemented DHA, and usually would supply 120–150 mg of DHA for each 20 g protein equivalent. Assessment of fatty acid status could be considered pre-conceptionally or early in pregnancy and supplementation can be given if biochemical deficiency is demonstrated.
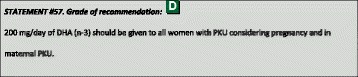


##### Nutrient monitoring

It is essential that key nutrients are monitored pre-conception and at the start of pregnancy, with further monitoring recommended only if there are concerns about dietary adherence or biochemical/clinical deficiency has been noted earlier in the pregnancy. On a non-supplemented low Phe diet, intake of vitamin B12 and a decreased intake of vitamin B12 may contribute to an increased risk of congenital heart disease [359]. If vitamin B12 status is low at the start of dietary treatment it should be corrected with oral or intramuscular vitamin B12. Low selenium concentrations throughout pregnancy have also been noted in women with maternal PKU [186], without selenium supplements added to their Phe-free L-amino acid supplements. The MPKUCS revisited 28 pregnancies born with CHD. They had significantly higher blood Phe, lower proline, valine, methionine, isoleucine, leucine, lysine, arginine and lower red blood cell folate [360].

It is important to note that interpreting micronutrient blood markers is challenging during pregnancy due to the maternal, placental and fetal adaptations, which vary between individuals and are dependent on gestational age. These issues lead to reduced sensitivity and specificity of biomarkers particularly during late pregnancy and target blood ranges used for non-pregnancy may be inappropriate during pregnancy [387].
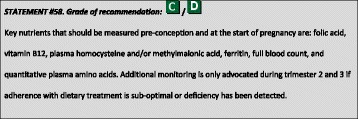


##### Breast-feeding and lactation

Unaffected infants of maternal PKU women are able to metabolize the Phe contained in their mothers breast milk without difficulty [102, 388] and women with PKU should be encouraged to breast-feed their infants. The only contraindication is if the mother is treated with BH4 [389] because the product characteristics state that it is unknown if the drug or its metabolites are excreted in human breast milk. However, we encourage breastfeeding and do not consider there are contraindications for breastfeeding in (maternal) PKU, even with BH4.

There is some suggestion that the Phe content of breast milk is higher than milk from healthy mothers. The Phe content of maternal breast milk is highest immediately post birth (up to 238 mg/100 ml) but decreases to 90 to 130 mg/100 ml [390]. Bradburn et al. reported the Phe content of breast milk was 86 mg/100 ml at day 6 post-partum and 74 mg/100 ml at day 13 post-partum [388].

There is no published data about breast-feeding infants with PKU if mothers also have PKU. However, practical experience would suggest that breast-feeding is possible providing the same management principles as for all infants with PKU (i.e. a Phe-free formula is given pre-breast feeds) [391] are adopted.

Lactation is an exceptionally demanding nutritional state for the mother. Factors relating to sub-optimal maternal nutrition status during lactation include maternal age, quality of dietary treatment, lifestyle factors, and spacing of consecutive births [392]. Energy requirements of milk production are high with energy requirements considered to increase by 505 kcal/day to 675 kcal/day in the first 6 months of breast-feeding [393]. It is assumed that part of extra energy requirement will be met by fat stores that are laid down during pregnancy. An additional 15 g/day (approximate amount) protein to pre- pregnancy requirements should be provided [394].

There are no reports detailing Phe intake during lactation, probably because many women discontinue strict diet after pregnancy [395]. Blood Phe concentrations are likely to rise significantly associated with post-partum catabolism unless dietary energy intake and a low Phe intake is maintained. Phe requirements are likely to remain similar to pre-pregnancy requirements. It is important women are encouraged to return to a healthy weight post pregnancy. All women should receive regular nutritional support post pregnancy, and women who have discontinued dietary treatment may be particularly vulnerable to the effects of poor food choices.
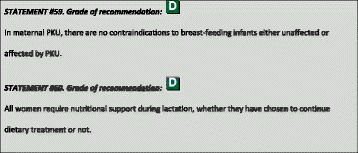


##### Dietary education programmes for maternal PKU

The low Phe diet is challenging for women with PKU during pregnancy and women need much support and education about maternal PKU treatment. Many women have followed a normal diet for years and may never have managed their own dietary treatment. In PKU, poor EF, e.g. sub-optimal planning and organizational skills, poor attention [70], and short-term memory [93], may affect the ability to self-manage a low Phe diet because of the day to day organization, technical skills and planning required [396]. Obtaining Phe-free L-amino acid supplements and low protein foods may be difficult. Poor dietary adherence was associated with the following maternal factors: younger women (25 and under), those with less formal education, (high school or less), and women using social assistance [397].

Women with a lower IQ require intensive practical help with dietary application. A ‘Resource Mother’ or diet support worker, providing practical assistance to women with PKU, has been proven to be very helpful by providing social support, enhancing positive attitudes toward the treatment and ensuring that necessary resources were in place [335]. This care is provided in patients own home. In a USA study, the Maternal PKU Resource Mothers Program matched mothers of children with PKU (Resource Mother) to women with PKU who were planning a pregnancy or who were already pregnant, with the aim of providing social support, enhancing positive attitudes toward the treatment and ensuring that necessary resources were in place. Women who received the services of a ‘Resource Mother’ attained metabolic control on average, 2 weeks sooner than women who did not participate [335]. Waisbren et al. [398] also found that strong social practical and emotional support from family and medical providers greatly increased the chance that a woman would start treatment before pregnancy.

Alternatively, some patients are admitted to the hospital for a 3–5 day intensive education. As well as receiving dietary and cooking advice, they are taught how to do their own blood Phe measurements [333].
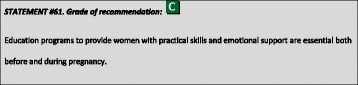


### Late diagnosed and untreated PKU

Several definitions are used to describe untreated, late diagnosed or late treated children and adults with PKU. In this section the terms **late diagnosed** and **untreated** PKU are used. **Late diagnosed** refers to children diagnosed between the ages of 3 months to 7 years (≥3 months - <7 years). **Untreated** PKU refers to patients untreated by 7 years of age and over. It is acknowledged that these definitions are arbitrary but improvement in IQ is rarely observed if treatment is started after the age of 7 years [399, 400].

There are many late diagnosed and untreated patients with PKU due to a lack of NBS, NBS failures and/or immigration of patients from countries without NBS or treatment [74, 401–404]. Some cases may not be diagnosed until adulthood, presenting with neurological complications (without severe neurocognitive impairment) possibly related to PKU [405, 406].

Untreated patients with severe intellectual disability and challenging behavioural problems have high support needs [407], and some may live in social welfare homes [408, 409]. An increase in life expectancy suggests the importance of their identification and the provision of long-term care planning [410]. Intervention with a low Phe diet may be beneficial [407]. Their overall rehabilitation program should not be different from individuals with other causes of intellectual disability.

### Late diagnosed PKU

Late diagnosed PKU patients may significantly benefit from the introduction of a low Phe diet, which may improve intellectual performance [74, 411, 412]. Outcome is mainly influenced by age and developmental quotient/IQ at the start of treatment [399, 400]. Reversibility of IQ loss may occur especially in the first 4–6 years of life [74, 399, 400], although this has also been reported in an 8 year old child [413].

#### Untreated PKU

Untreated PKU patients even with severe intellectual disabilities may benefit from the introduction of a low Phe diet. Several case reports and cohorts describe changes in symptoms in untreated adults after commencement of dietary treatment [71, 399, 401, 413–426], although benefit is not seen in all patients [71, 414, 415, 418, 422, 424, 426, 427]. Mainly improvement of motor function (tremors/spasticity) and behaviour (less restless and irritable, more alert/responsive and less aggressive with decreased numbers and severity of self-injury behaviours) are described (Table 16). Giffin et al. [428] reported improvement in visual attention span in 2 out of 3 patients. Schuett et al. [429] recounted positive and negative results after diet commencement in 42 mildly and severely intellectually impaired patients concerning outcomes such as mood, hyperactivity, body weight and nausea/vomiting.

**Table 16:** Expected positive outcome changes with Phe-restricted dietary treatment in untreated PKU patients

Behaviour	
Less aggressive behaviour, self-injury, hyperactivity, restlessness, irritability, sleep disorders, anxiety, stereotyped behaviour	
Improved mood change, social interaction, verbal communication, daily living skills	
Neurology	
Improvement of attention span, alertness, short-term memory processes, motor skills, seizures, spasticity, tremors	
Other clinical parameters	
Improvement/disappearance of eczema, skin rash, body odour Darker hair colour	
Quality of life	
Reduced nursing time	
Medication	
e.g. less use of sedative, anti-psychotic, anticonvulsants	

Reported in the literature of chapter 9.2 untreated PKU

Brown et al. [430] determined that a low Phe diet in adults with previously untreated PKU resulted in economic benefit to the health service and society in general. Reduction in nursing time, hospitalizations, outpatient clinic visits and medications reduced mean annual costs [430].

#### Dietary treatment and monitoring

Before diet initiation, it is essential to consider the individual patient and their quality of life especially if they have severe intellectual disability and/or behavioural problems [74]. A low-Phe diet for previously untreated adults should be supervised by a team experienced in the treatment of PKU. Care providers, family members and/or residential house staff members require instruction about the practicalities of dietary treatment. Potential management barriers should be identified. Care providers and families may consider that the diet is too restricted and so inappropriate [417]. Clear information should be given about the potential benefits of dietary treatment. A practical plan for stepwise introduction of a low Phe diet is published by Dolan et al. [417], and Hoskin [431]. Dietary management needs careful nutritional monitoring to optimize Phe, total protein energy and micronutrient intakes. Adjustment of Phe-free L-amino acid supplements may be necessary [127]. When treatment does improve patient behaviour and social interaction, adjustment of social and therapeutic programs is essential. Potential expected outcome changes are identified in Table 16.

Although not fully understood, in some patients post diet initiation, there may be deterioration of symptoms with increased frequency of aggressive behaviour and recurrence of seizures [426]. It may be necessary to consider discontinuation of dietary management. It is not possible to predict which patients will respond to a low Phe diet and the first clinical or behaviour changes may not occur for weeks or even months. However, diet discontinuation should be considered only if there is no clinical or behavioural improvement after 6 months, providing blood Phe levels have consistently been maintained within target range. Videotaping can help to record changes in behaviour.

Phe monitoring is recommended weekly at treatment commencement but should follow standard recommendations for monitoring once blood Phe levels have stabilised within target range. Target Phe levels and nutritional follow-up recommendations are discussed in the chapter treatment goals and follow up. Although it is not clear what the optimal target blood level should be in untreated adult patients, we recommend <600 μmol/l. Dolan et al. [417] and Koch et al. [399] reported upper target Phe levels of 600 μmol/l [417] and 720 μmol/l [399] respectively.

#### Other therapies

There are limited reports on the use of additional therapies in later or undiagnosed PKU. Kalkanoglu et al. [432] demonstrated in a double-blind cross-over study improved concentration, awareness and less self-injurious behaviour in 14 of 19 untreated adults by LNAA supplementation. Vernon et al. [433] reported one BH4-responsive untreated adult with PKU with a baseline plasma Phe of 1255 μmol/l. On BH4 treatment he showed significant behavioural improvements, resulting in fewer behavioural problems and increased social interactions. Treatment with BH4 should be considered for any responsive patient independent of their mental capacity. BH4-treatment is discussed in the chapter pharmacological treatment and emerging therapies.
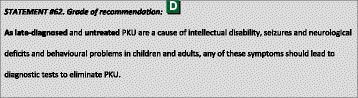
 
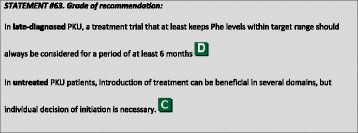


### Adherence

Poor treatment adherence is common in any chronic disease. Counselling and education is often recommended to improve adherence. Research indicates that whilst knowledge is necessary for adherence, it is not a strong predictor of adherence [198, 434]. Short-term gain in knowledge can be achieved by intervention programs such as summer camp, but adherence and metabolic control do not improve after interventions [435–437]. Changes in attitude and motivation may be more effective [438].
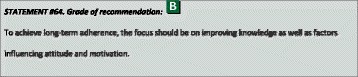


Whereas traditionally the metabolic team prescribed and monitored treatment in PKU, this is now commonly done in partnership with the caregivers and patients. It is recognised that it is valuable to provide person-centred care, providing advice and support that is focussed on the individual rather than being judgmental about poor adherence [10]. This is particularly important with adolescents and adult patients. Adult patients often prefer management that interferes with normal life as little as possible. They are expected to take responsibility for their own health with the metabolic team providing the right information, tools and motivational encouragement [10]. Unfortunately, some adult patients no longer attend hospital appointments for follow-up care, and one of the most difficult challenges is re-engagement of this patient group. They may have poor understanding of their condition, its potential consequences and retain unpleasant memories of dietary treatment. Reaching out to this cohort necessitates collaborative efforts of PKU clinics and national patient advocacy organizations, possibly using social media, to help nurture them to return to clinical follow-up [439].

In children under 12 years of age, although uncommon, there are cases in which parents or caregivers refuse to engage with healthcare professionals. Common signs of poor adherence in early childhood include: persistently poor Phe control, failure to engage with health professionals (e.g. non-attendance at clinic appointments, non-response to telephone calls), and sporadic return of blood Phe spots [440]. Known factors associated with chronic poor adherence that affects parenting capacity includes lack of intellectual abilities of parents, alcoholism, drug abuse and mental health issues [441] and other social issues including financial issues and chronic illness of parents. Health care providers have a legal obligation to protect and care for each child in their clinic and recommendations for action when a child has chronic poor metabolic control are summarised in statement #65:
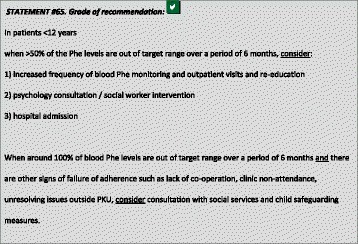


### Pharmacological treatment and emerging therapies

#### BH4 treatment

BH4, also known as sapropterin dihydrochloride (the active compound in the commercial drug) is used to treat a subset of PKU patients with PKU [193, 311, 433, 442–453]. Patients with high residual activity of the PAH enzyme have a greater probability of BH4 response, but a minority of patients with classical PKU also may benefit from BH4 treatment [193, 194, 442, 443, 445, 449, 453]. Recently, efficacy and safety of BH4 has been demonstrated in children <4 years of age which has led to European approval for BH4 in this age category [454–457]. BH4 is still unavailable in some European countries.

Two systematic reviews have summarised the efficacy and safety of BH4. Somaraju et al. [458] reviewed 2 randomised controlled trials that were led respectively by Levy et al. [447] and Trefz et al. ([459], whereas Lindegren et al. [448] included the following additional studies/reports: one uncontrolled open-label study, one prospective cohort study, and several case series. Both systematic reviews concluded there is short-term evidence to demonstrate that BH4 is effective in reducing blood Phe concentrations and increasing Phe tolerance in BH4-responsive PKU patients. Also no serious adverse events were reported. Several uncontrolled open-label studies and case series support a significant reduction of blood Phe levels [193, 194, 311, 433, 442, 444–451, 453–455, 458–464] and increased Phe tolerance [193, 194, 311, 433, 443–446, 448–452, 454, 455, 458, 459, 463–465]. These benefits have also been replicated in longer-term (investigated up to 5 years) studies [193, 194, 443, 445, 446, 451, 454, 455, 463, 465]. Furthermore, less variability in blood Phe control has been described in 3 descriptive papers [311, 454, 460]. Current data suggests that with BH4 treatment cognition and behaviour issues may improve but, possibly, more importantly they do not deteriorate [155, 183, 466]. The same applies for quality of life [58, 165]. At present, studies have not reported long-term neurocognitive outcome, behaviour and quality of life with BH4 treatment.

The cost-effectiveness of BH4 is not established, especially when dietary treatment and Phe-free L-amino acid supplements are still required.

BH4-responsiveness should be determined on an individual case basis. The degree of responsiveness will be characterised by the extent of improvement in biochemical control and increase in natural protein intake. We define BH4-responsiveness as ‘*establishing an increase in natural protein tolerance of ≥100% with blood Phe concentrations remaining consistently within the target range’*. BH4-responsiveness can also be defined by improved metabolic control ‘*>75% of blood Phe levels remaining within target range without any decrease in natural protein intake associated with BH4 treatment’*. BH4 should only be prescribed in cases of proven BH4-responsiveness which is established by a treatment trial (chapter 11.2). BH4 should be withdrawn if blood Phe levels consistently exceed the upper target range and there is no improvement associated with any increase in BH4 dosage. If nutritional status deteriorates e.g. obesity or development of nutritional deficiencies, discontinuation of BH4 treatment should be considered.
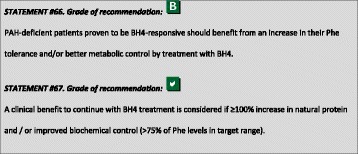


##### BH4 and pregnancy

Since drug studies in pregnancy are not feasible, experience is based on a small number of case reports and 2 small cohort studies. Therefore, no prospective data is available regarding the indication, dose and management of BH4 during pregnancy. In a European cohort study, 3 patients received BH4 pre-conception, and 5 patients commenced treatment during pregnancy [467]. In a USA cohort study, 15 patients were administered BH4 prior to pregnancy, and only 1 patient received BH4 post-conception [468, 469]. Overall, the dosage varied between 4 and 20 mg per kg of body weight. The Kuvan® Adult Maternal Paediatric European Registry reported 4 pregnancies, with varying BH4 doses between 3 and 17 mg per kg of body weight. No foetal development problems or adverse events related to the pregnancies were observed [465].

Case descriptions show that BH4 assists in lowering blood Phe levels to within target range. All the infants exposed to BH4 during pregnancy had favourable outcomes, except in 1 case when the mother had very high Phe levels in early pregnancy and was given BH4 as a rescue treatment. BH4 treatment can be given during pregnancy, but only if women are known to be BH4-responders and dietary treatment alone is unsuccessful in achieving target blood Phe control. Potential responsiveness can be assessed by genotyping and/or a BH4 loading test [35].
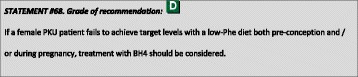


##### BH4 and untreated adults

This is discussed in the chapter on untreated adults.

#### BH4 loading test and treatment trial

Before treating patients with BH4, assessment of BH4-responsiveness is essential. Determination of BH4-responsiveness can be done by genotype and/or BH4 loading tests. As already reported in chapter 4.3, the genotype may predict or exclude to some degree BH4-responsiveness [32–34]. Every patient except if there are 2 null-variants justifies a BH4 loading test. If 2 BH4-responsive variants are identified, a treatment trial without a BH4 loading test can be commenced.

The short-term loading test take up to 48 h in Europe and 28 days in USA, whereas the treatment trial in potential BH4-responders (after a positive short-term test or genotype in line with known BH4-responsiveness) may occur over a few weeks or even months [470]. Bernegger et al. [471] described a 24 h BH4 loading testing using 20 mg/kg to differentiate between BH4-responders and non-responders. Extension to 48 h and repeated BH4 administration seems to be useful to detect slow responders and responsiveness in more severe phenotypes [472]. The utility of a 48 h test has been confirmed in a study of 177 patients treated with 20 mg BH4/kg/day [35].

At present, the exact procedure of the BH4 loading test differs among countries, also depending on laboratory availability. Blau et al. [470] and Singh et al. [450] published protocols for BH4 loading tests. The 24 h test in newborns can detect BH4-deficiencies in addition to BH4-responsiveness in PAH patients [473]. The arbitrary responsiveness definition of a > 30% reduction in blood Phe appears to be a good compromise between sensitivity and specificity for the initial screening test. Individual patient characteristics should be considered when interpreting results, especially in patients with low baseline Phe levels [474].

Every positive BH4 loading test and gene variants analysis refers to a potential BH4-responsiveness. Long-term response should be proven in a treatment trial adjusting the BH4 dosing, natural protein intake and Phe-free L-amino acid supplements. The starting dose in a treatment trial is 10–20 mg per kg of body weight and can be adapted during the trial. This process may require several weeks to months. There is a lack of studies that have addressed the long-term treatment BH4 dose, the natural protein tolerance and supplementation with Phe-free L-amino acids. When adapting diet with BH4, Singh et al. [450] advised to increase the natural protein first, followed by reduction in Phe-free L-amino acid supplementation. An additional step should include a reduction in the BH4 dose. It is important to maintain blood Phe concentrations in the target range with BH4 treatment and diet relaxation. It is also important to anticipate that patients may not always make the healthiest food choices when given some dietary freedom. Increasing the natural protein (from non-animal food sources) and decreasing the Phe-free L-amino acid supplementation may even result in nutritional deficiencies [75, 452].
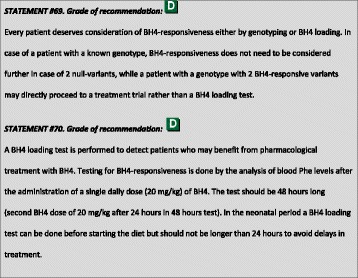


#### Emerging therapies

Effectiveness of PKU treatment is demonstrated by any of the following objective measures: reduction in Phe blood concentrations, increase in natural protein tolerance, improvement in neuropsychological testing, improved nutritional staus and better quality of life. Special considerations should be made for patients at different ages and special situations such as pregnancy or breast-feeding.

A possible enzyme replacement therapy using PEG-Phenylalanine-Ammonia Lyase (PAL) or Pegvaliase is under investigation. PEG-PAL clinical phase II trials have proven short-term reduction in the Phe blood concentrations in adult PKU patients, but further studies are required to observe long-term effectiveness and safety [475]. Results of a phase III extension study (NCT01819727) are awaited. Gene therapy and therapeutic liver repopulation are being investigated in murine models only [476, 477], and larger animal PKU models and human studies are being developed.

### Patients’ view

There are several papers discussing patients’ and parents’ coping strategies and barriers to effective management. Awizus et al. [478] interviewed 11 parents of children aged 8 years with PKU. Parents were shocked by the diagnosis, had emotional and adjustment problems and were commonly in conflict between the task of attaining acceptable blood Phe levels and the guilt they experienced when they deviated from the dietary rules to meet their children’s demands. In another study parents described their children with PKU as feeling different from peers [479] whereas patients described themselves as ‘healthy’ or ‘normal’ [480, 481]. Di Commo et al. [480] concluded from 20 patients with PKU, that they consider PKU more as a potential risk rather than an actual disease and that adherence seems connected with highly internalized behaviour rather than with perception of adverse effects. Some PKU patients avoid social occasions where food is shared because of fear of stigmatization. Patients (*n* = 47) experience a paradox, either they feel normal but isolated from the social context, or are different while participating in the convivial aspects of the social settings [481].

Primary obstacles to successful management of PKU identified by parents of 32 PKU children were time constraints, stress associated with food preparation, record keeping, and social life restrictions imposed by PKU. About two-thirds agreed that a home-monitoring blood device was desirable to ease the burden of management [482].

Bernstein et al. [483] studied the effectiveness of educational tools perceived by clinicians, parents and patients. They found a discrepancy between patient and clinician views regarding the effectiveness of nutrition education. Patients concluded that their families were the most effective educators whilst parents responded they felt one-on-one counselling was the most effective educational tool [483].

Hagedorn et al. [11] reported the minimum standard of care requested by patients/caregivers and the chief requirements were: uniform treatment/management goals; care by a multidisciplinary team (physician, dietician/nutritionist and psychologist); and access to care, BH4, and special dietary products [11].

In order to represent the interest of patients, national patient organizations are recommended.

### Implementation/impact of guidelines

Distributing the European PKU guidelines may increase awareness of their presence, but in itself will generally not lead to behaviour change [484]. Interactive educational interventions and reminders (when used sparingly) to health care providers are considered to be effective [484]. Potential barriers to behaviour change are lack of motivation, inadequate facilities and resources. The impact of these guidelines on daily care will differ among countries. Change of target blood Phe levels and the recommendation to follow up and treat patients for life may have impact on the intensity of care. The same applies to other recommendations such as the follow up of bone mineral density, nutritional status, neurocognition and frequency of Phe measurement and outpatient clinic visits.

Some recommendations can have impact on the health care budget. For example, in some centres there may be a need for additional staff in their paediatric healthcare team and/or for transition to adulthood. Reimbursement for BH4 treatment is not established in all countries.

The aim is to have the entire guideline updated in 5 years. The actual impact of the guidelines on change in healthcare will be evaluated by questionnaires.

### Future requirements and research

It is evident that many of these guideline statements have not yet been introduced into clinical practice by several European centres and it is also clear that various barriers, including financial hurdles, may impede the speed of change. Unmet needs include identification and training of sufficient numbers of physicians with a broad interest in co-ordinating care for adult patients with IMD including PKU. We need to optimize PKU treatment both in adult and elderly care. The development of a device able to accurately measure and generate immediate blood Phe results for home monitoring (instead of home sampling) is likely to change management practices. It will decrease metabolic laboratory time, and will dramatically reduce the time between blood sampling and obtaining a blood Phe result, and so assist and motivate patients to achieve target blood Phe target ranges more easily. The establishment of the expert reference network (ERN) may lead to patient treatment being directed by European designated expert centres with ‘local’ care provided by experienced treatment teams and may facilitate the use of international databases by which studies on larger populations can be performed. There is an ongoing need for meta-analysis relating outcome to metabolic control during childhood, adolescence, and adulthood, while the need for more sophisticated statistics in such studies is underestimated.

Future research in PKU may target the pathophysiology of brain dysfunction aiming to improve treatment strategies. These strategies may not only target the blood Phe concentrations, but also directly alter cerebral metabolism. They will aim to improve neuropsychological outcome and functioning as well as provide a better quality of life by decreasing the need for arduous dietary Phe restriction. Such treatments may include new drugs such as enzyme replacement therapy using PEG-Phenylalanine-Ammonia Lyase (PAL) but other non-nutritional treatment options including gene therapy and therapeutic liver repopulation have not progressed beyond animal models.

Future research is necessary to identify the number of adults who experience clinical symptoms together with better characterisation and impact of sign and symptoms. More data is needed about the influence of metabolic control during adolescence and adulthood, particularly when childhood metabolic control is optimal. In addition, new strategies should be actively sought to re-engage adult patients who are no longer in active hospital follow up but who are at risk of mental health and executive function deficits.

Other future research topics include strategies to improve adherence particularly in adolescents and adulthood, efficacy of enzyme replacement therapy, effectiveness of GMP and LNAA, usefulness of biochemical markers such as Phe variability, the Phe: Tyr ratio as well as the ratio of Phe to other LNAA, new (long-term) biomarkers, defining the optimal lower target Phe levels, bioavailability of micronutrients in Phe-free L-amino acid supplements and the functionality and long-term side effects of Phe-free L-amino acid supplements in PKU.

## Conclusion

These first European guidelines are the result of a 3 year process based on the AGREE and SIGN methodology as PKU management differs accros Europe. The level of evidence of most recommendations is C or D. Although study designs and patient numbers are sub-optimal, many statements are convincing, important and relevant, and may set the benchmark for improving outcome in PKU patients. Key recommendations which should be prioritised for implementations mainly relate to treatment initiation, target Phe levels for treatment, and follow-up. Minimum requirements regarding management and follow-up of PKU patients are formulated. Knowledge gaps are identified that require further research in order to direct better future care. Future research should focus on the pathophysiology of brain dysfunction aiming to improve treatment strategies and the impact of metabolic control during adolescence and adulthood. These guidelines are aimed to standardize care and do determine a course of action, but are not mandatory. The authors of these guidelines are willing to update these guidelines based on the highest quality evidence available.

### Definitions

**Table:** 

Concurrent Phe level	The Phe level measured at or close to the day of outcome assessment.
ESPKU	The European Society for Phenylketonuria and Allied Disorders is the umbrella organization of national and regional associations from about 30 countries established by parents.
Executive functioning	Executive functioning are cognitive processes that regulate behaviour; examples are inhibitory control, working memory and cognitive flexibility.
High risk pregnancy	A pregnancy that threatens the health or life of the mother or her foetus.
Late treated and/or untreated adults	Late diagnosed refers to children diagnosed aged between 3 months and 7 years (≥3 months - <7 years). Untreated refers to patients untreated aged 7 years and over.
Lifetime Phe levels	Phe levels from birth to the age of testing. This is often expressed as the median Phe level of (semi)annual mean Phe levels. Also referred to as historical Phe levels.
Maternal PKU syndrome	The teratogenic effects of elevated maternal phenylalanine levels during pregnancy to the foetus.
Phenylalanine tolerance	The amount of phenylalanine (mg/kg/day or mg/day) that maintains plasma phenylalanine concentrations within the target range. This may also be described as natural protein tolerance expressed as g/day taking a phenylalanine content in natural protein as 50 mg phenylalanine/g natural protein.
Protein requirements	The lowest level of dietary protein intake that will balance the losses of nitrogen from the body, and thus maintain the body protein mass in persons at energy balance with modest levels of physical activity.
Protein substitutes (phenylalanine-free L-amino acid supplements and low phenylalanine glycomacropeptide protein)	Protein replacement/substitutes are essential to prevent protein deficiency and optimize metabolic control. Protein substitutes are mainly sourced from phenylalanine-free L-amino acids supplements and less commonly from low phenylalanine glycomacropeptide.
Potential tetrahydrobiopterin (BH4) responsiveness	>30% reduction in blood phenylalanine in a BH4 loading test or 2 BH4 responsive variants. Long-term BH4-responsiveness should be proven in a treatment trial adjusting the BH4 dosing, natural protein intake and phenylalanine-free L- amino acid supplement.
Tetrahydrobiopterin (BH4)	Cofactor of the phenylalanine hydroxylase. BH4 also acts as a chaperone molecule in phenylalanine hydroxylase-deficient patients harbouring specific gene variants. BH4 is also a cofactor of tyrosine and tryptophan hydroxylases and plays an important role in the conversion of L-arginine to nitric oxide (NO) by nitric oxide synthases (NOS).
Tetrahydrobiopterin (BH4) deficiencies	Defects in BH4 metabolism (either synthesis or regeneration) result in a deficiency of BH4. These include 3 known defects for synthesis and 2 for regeneration.
Tetrahydrobiopterin (BH4) responsiveness	In this report it is defined as an increase of ≥100% in natural protein and/or improved biochemical control (>75% of phenylalanine levels in target range) on a dose of BH4 that ranges between 1 and 20 mg perkg of body weight (with a maximum dose of 1000 or 1400 mg/day in some cntries).

## Abbreviations

ADHD: Attention deficit hyperactivity disorder
AGREE: Appraisal of guidelines for research and evaluation
APL: Assigned blood Phe levels
BDI: Beck depression inventory
BH4: Tetrahydrobiopterin
BMD: Bone mineral density
BMI: Body mass index
CBCL: Child behaviour checklist
CHD: Congenital heart disease
DACH-RDA: Regular daily allowance recommended for the German speaking countries Germany, Austria and Switzerland
DBS: Dried blood spots
DHA: Docosahexaenoic acid
DHPR: Dihydropteridine reductase
DXA: Dual-energy X-ray absorptiometry
EF: Executive function
EPA: Eicosapentaenoic acid
ERN: Expert reference network
ESPKU: European Society of Phenylketonuria and Allied Disorders
ETPKU: Early treated PKU
FAO: Food and Agriculture Organization of the United Nations
GMP: Glycomacropeptide
GTPCH: GTP cyclohydrolase I
HPA: Hyperphenylalaninemia
HRQoL: Health related quality of life
IMD: Inherited metabolic disorder
IQ: Intelligence quotient
ISCD: International Society for Clinical Densitometry
IUGR: Intrauterine growth retardation
LC-PUFA: Long chain polyunsaturated fatty acids
LNAA: Large neutral amino acids
MBD: Mineral bone disease
MHP: Mild hyperphenylalaninemia
MPKUCS: Maternal PKU collaborative study
MRI: Magnetic resonance imaging
NBS: Newborn screening
PAH: Phenylalanine hydroxylase
PAL: Phenylalanine ammonia lyase
PCD: Pterin-4a-carbinolamine dehydratase
PEG: Polyethylene glycol
Phe: Phenylalanine
PKU: Phenylketonuria
PTH: Parathyroid hormone
PTPS: 6-pyruvoyl-tetrahydropterin synthase
RDA: Recommended dietary allowances
RNI: Reference nutrient intakes
SIGN: Scottish intercollegiate guidelines network
SR: Sepiapterin reductase
Tyr: Tyrosine
UNU: United Nations University
USA: The United States of America
WHO: World Health Organization
WM: White matter
WMA: White matter alteration
